# *ERBB2* in Cat Mammary Neoplasias Disclosed a Positive Correlation between RNA and Protein Low Expression Levels: A Model for erbB-2 Negative Human Breast Cancer

**DOI:** 10.1371/journal.pone.0083673

**Published:** 2013-12-26

**Authors:** Sara Santos, Cláudia S. Baptista, Rui M. V. Abreu, Estela Bastos, Irina Amorim, Ivo G. Gut, Fátima Gärtner, Raquel Chaves

**Affiliations:** 1 Institute for Biotechnology and Bioengineering, Centre of Genomics and Biotechnology, University of Trás-os-Montes and Alto Douro, Vila Real, Portugal; 2 Veterinary Clinics of University of Porto, Institute of Biomedical Sciences Abel Salazar, University of Porto, Porto, Portugal; 3 CIMO-ESA, Instituto Politécnico de Bragança, Bragança, Portugal; 4 Department of Genetics and Biotechnology, School of Life Sciences and Environment, University of Trás-os-Montes and Alto Douro, Vila Real, Portugal; 5 Institute of Pathology and Immunology, University of Porto, Porto, Portugal; 6 Department of Pathology and Molecular Immunology, Institute of Biomedical Sciences Abel Salazar, University of Porto, Porto, Portugal; 7 Centre National de Genotypage, Evry, France; University of Alabama at Birmingham, United States of America

## Abstract

Human *ERBB2* is a proto-oncogene that codes for the erbB-2 epithelial growth factor receptor. In human breast cancer (HBC), erbB-2 protein overexpression has been repeatedly correlated with poor prognosis. In more recent works, underexpression of this gene has been described in HBC. Moreover, it is also recognised that oncogenes that are commonly amplified or deleted encompass point mutations, and some of these are associated with HBC. In cat mammary lesions (CMLs), the overexpression of *ERBB2* (27%–59.6%) has also been described, mostly at the protein level and although cat mammary neoplasias are considered to be a natural model of HBC, molecular information is still scarce. In the present work, a cat *ERBB2* fragment, comprising exons 10 to 15 (*ERBB2*_10–15) was achieved for the first time. Allelic variants and genomic haplotype analyses were also performed, and differences between normal and CML populations were observed. Three amino acid changes, corresponding to 3 non-synonymous genomic sequence variants that were only detected in CMLs, were proposed to damage the 3D structure of the protein. We analysed the cat *ERBB2* gene at the DNA (copy number determination), mRNA (expression levels assessment) and protein levels (in extra- and intra protein domains) in CML samples and correlated the last two evaluations with clinicopathological features. We found a positive correlation between the expression levels of the *ERBB2* RNA and erbB-2 protein, corresponding to the intracellular region. Additionally, we detected a positive correlation between higher mRNA expression and better clinical outcome. Our results suggest that the *ERBB2* gene is post-transcriptionally regulated and that proteins with truncations and single point mutations are present in cat mammary neoplastic lesions. We would like to emphasise that the recurrent occurrence of low erbB-2 expression levels in cat mammary tumours, suggests the cat mammary neoplasias as a valuable model for erbB-2 negative HBC.

## Introduction

Several aspects contribute to the value of domestic animals as models for human cancers [1], [2]. Also, it is generally recognised that the use of animal models for the safety testing of investigational drugs is imperfect and needs more accurate (predictive) preclinical animal model screening that has the potential to increase the pace and reduce the cost of successful drug development for breast cancer [3]. Similarities in both histology and biological behaviour support the idea of cat mammary primary malignant lesions (MaLs) as a possible model to study HBC [4]. In fact, several authors consider that spontaneous cat mammary pre-invasive intraepithelial lesions (hyperplasias and neoplasias) and malignant lesions share the full spectrum of morphological features with their human counterparts [2]–[5]. The annual incidence of cat mammary neoplasia has been estimated at 12.8–25.4 per 100.000 female cats, and mammary malignant neoplasia represents an important cause of cat mortality. The incidence and morbidity of these tumours have been demonstrated to be very high due to their rapid growth, high proliferation rates, and capability to metastasise to regional lymph nodes and distant organs [6]–[9].

The pathogenesis and progression of invasive breast cancer have been related to a large variety of growth factors, and the proteins of the epithelial growth factor receptor family have been the most investigated in this heterogeneous disease [10], [11]. The *EGFR* family includes four receptors: *EGFR* (or *ERBB1*), *ERBB2* (also known as *NEU*, *EGFR2* or *HER2*), *ERBB3*, and *ERBB4* [12]–[15]. These receptors share an overall structure with an extracellular region, a transmembrane region and an intracellular carboxy-terminal tail that contains a tyrosine autophosphorylation site [16], [17]. The activation of oncogenes by mutation or copy number gain (DNA amplification), along with the loss ofactivity of tumour suppressor genes by mutation or copy loss, are somatic alterations of the genome that result in tumour initiation [18]–[20]. Also, it is highly recognised that acquired somatic mutations are responsible for approximately 90% of breast tumours [21]. In summary, genes that are commonly amplified or deleted often include point mutations that activate or inactivate the oncogenes [22], [23]. It is important to point out that the activation of oncogenes, such as *ERBB2*, provides an opportunity to develop therapeutic targets of the affected protein itself or for a downstream event [20]. The monoclonal antibody trastuzumab (Herceptin®), which targets the activated oncogenic forms of erbB-2 protein and acts as a kinase inhibitor, was developed and approved for use as an HBC treatment [24]–[27].

In recent years, a systematic investigation of the potential role of inherited germline variants of *ERBB2* in breast cancer risk has been conducted using single nucleotide variants and haplotype-based analyses [28]–[32]. The most notable overall observation in this investigation is the lack of evidence to support a significant association between *ERBB2* genomic sequence variants (SVs) and HBC initiation, despite the wealth of information supporting its role in breast cancer progression [16], [31], [32]. Concerning the determination of breast cancer prognosis, it has recently been suggested that the quantification of *ERBB2* mRNA transcripts by qRT-PCR should be applied to the routine erbB-2 IHC procedures as an additional molecular test [33], [34]. In previous studies, the authors state that a fraction of human breast cancers [35]–[37] and cat mammary lesions [38] show a good correlation between high expression levels of *ERBB2* mRNA and the erbB-2 protein. In more recent works, low *ERBB2* RNA expression levels have been described in HBC, suggesting the underexpression of this gene [39]–[40]. In cat mammary lesions, alterations of the *ERBB2* proto-oncogene have been studied, mostly at the protein level, and, as occurs in HBC, the overexpression of the erbB-2 protein has been recognised to confer poor prognoses to CMLs [4], [38], [41]–[43]. Although cat mammary neoplasias are considered to be a natural model of human breast cancer, molecular information on benign and malignant lesions, particularly regarding the cat *ERBB2* gene and erbB-2 protein, is still scarce [44].

In the present work, we characterised the cat *ERBB2* gene in normal samples and cat mammary lesion samples by different approaches in order to obtain novel information concerning the *ERBB2* gene in the cat mammary tumour system (genome and proteome). Our study focuses on the cat *ERBB2* DNA fragment from exon 10 to 15 (*ERBB2*_10-15), which codes for the part of the extracellular domain of the erbB-2 protein that is targeted by the therapeutic antibody trastuzumab (Herceptin®).

The present analysis includes a collection of non-neoplastic and neoplastic lesions that represent a morphologically and clinicopathologically heterogeneous group of samples. Different approaches were employed, such as comparative studies of the cat *ERBB2* DNA sequence with its human counterpart, sequence variant characterisation, DNA and mRNA status evaluation by qRT-PCR, protein level quantification by immunohistochemistry (with two different antibodies), *in silico* coding sequence and respective translated protein construction, and determination of the consequences of the identified exonic non-synonymous sequence variants (nsSVs) in the cat erbB-2 3D structure. We also analysed and correlated the results obtained by these different techniques with the clinicopathological traits of the cat mammary neoplastic and non-neoplastic lesion samples.

## Results

A total of 43 cat mammary lesion samples and 23 normal samples (14 blood and 9 normal mammary gland samples) were collected for this work. All of the cat mammary lesions analysed in the present work were clinically and histologically characterised (Table S1). They included a collection of non-neoplastic and neoplastic lesions that represented a morphologically and clinicopathologically heterogeneous group of samples and were obtained over a two-year follow-up period (in the case of queens bearing malignant tumours). For statistical purposes, we established a tumour scale for clinical and histological grading and prognostic factors (Table S2).

### Analysis of the cat *ERBB2*_10/15 fragment in normal samples

**Figure 1:**
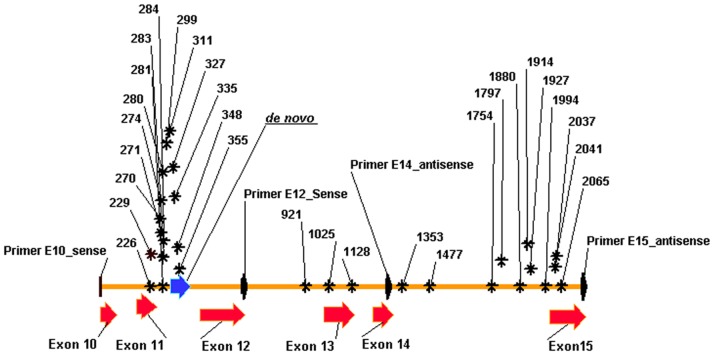
*In silico* physical map of cat *ERBB2* gene from exons 10 to 15 (*ERBB2*_10–15). Physical mapping of exons 10 to 15 (red arrows) and the *de novo* sequence genomic DNA (blue arrow; 84nt from 315–398 bp) corresponding to part of intron 11 of the cat *ERBB2* gene. The position of the four primers are point out in black arrows. The sequence variants detected in the present work are illustrated by its position nucleotide number and a black asterisk. The distribution of the genomic SVs detected is not homogeneous and they are predominantly localized in intron 11.

The cat *ERBB2* DNA fragment from exon 10 to 15 was amplified by polymerase chain reaction (PCR) with primers E10 and E15, using genomic DNA obtained from normal samples (blood) as template. After cloning and sequencing the amplified fragment, a final cat gDNA *ERBB2*_10/15 consensus sequence of 2173 bp was obtained. The gDNA *ERBB2*_10/15 sequence showed an incomplete alignment with the cat *ERBB2* RNA sequence (GenBank: AY702651.1), the cat Ensembl GeneScaffold 1508 and LGD gene-2064 (an *ERBB2* gene in the Genome Annotation Resource Field (GARField browser; *Felis catus* v12.2). Additionally, high similarity was detected between cat *ERBB2*_10/15 and its human counterpart (GenBank: NG007503; Figure S1). These preliminary result allowed us to recognise a *de novo*, 84 nt sequence (from 315–398 nt, comprising part of intron 11) and to identify the exon boundaries in the cat *ERBB2*_10/15 sequence (Figure 1).

### Sequence variant detection and prediction of coding regions and protein sequence

To search for *ERBB2*_10/15 sequence variants, two fragments, approximately 1300 bp (primers E10/E14) and 1500 bp (primers E12/E15) in length, were amplified by PCR. The sequences obtained from 14 normal gDNA samples (extract from blood samples) were multi-aligned, and a final consensus sequence was established (2173 bp), submitted to GenBank (reference JQ284376), and used in the present work as the reference or wild-type (wt) DNA sequence (cat *ERBB2*_10-15 sequence; Figure S1). All the sequences obtained for normal sample were multi-aligned with the *ERBB2*_10-15 wt sequence and 21 SVs were detected (Figure 1 and Table S3).

The same procedures were applied to the 19 gDNAs extracted from the CMLs, including 2 benign non-neoplastic (hyperplasia), 2 benign neoplastic, 12 primary malignant (MaL) and 3 metastatic lesions. The *ERBB2*_10/15 wt sequence was multi-aligned with the sequences obtained for each CML and 24 SVs were identified (Figure 1 and Table S3).

Some interesting results were obtained in the analysis of the 30 SVs detected, such as the fact that some SVs were only detected in one type of CMLs (Table S3). Moreover, the *in silico* physical mapping of the genomic sequence variations showed 7 SVs in exonic positions; two were synonymous variations (g.1025T>C and g.1128T>C) and 5 were non-synonymous variations (nsSVs; Table 1 and Table S3). Furthermore, from the 5 nsSVs, 1 was only detected in the normal samples (g.226 G>A) and 4 were only detected in the mammary lesion samples (Table S3).

**Table 1:** Frequent haplotypes detected independently in normal, mammary lesions and in total groups of samples.

Genomic SVs and Haplotypes																																
*SV Position*	*226*	*229*	*270*	*271*	*274*	*280*	*281*	*283*	*284*	*299*	*311*	*327*	*335*	*348*	*355*	*921*	*1025*	*1128*	*1353*	*1477*	*1754*	*1797*	*1880*	*1914*	*1927*	*1994*	*2037*	*2041*	*2065*			
*SV Alleles*	G>A(*1)	T>A(*2)	*T>G*	*(#)*	*G>T*	*G>A*	*G>C*	*del C*	*T>C*	*G>A*	*G>A*	*T>G*	*C>T*	*G>A*	*G>A*	*G>C*	T>C(*3)	T>C(*4)	*T>C*	*G>A*	*A>C*	*C>T*	*C>T*	*G>C*	*G>C*	*del A*	G>C(*5)	A>C(*6)	T>C(*7)			
*Haplotype Frequency*																														*Normal*	*CML*	*Total*
1	G	T	T	T	G	G	G	C	T	G	A	T	C	G	G	G	T	T	T	G	A	C	C	G	G	—	G	A	T	*0.167*		*0.094*
2	G	T	T	T	G	G	G	C	T	G	G	T	C	G	G	G	T	T	T	G	A	C	C	G	G	A	G	A	T	*0.125*		
3	G	T	T	T	G	G	G	C	T	G	G	G	C	G	G	G	T	T	T	G	A	C	C	G	G	A	G	A	T	*0.125*		*0.156*
4	G	T	T	T	G	G	G	C	T	G	A	G	C	G	G	G	T	T	T	G	A	C	C	G	G	A	G	A	T	*0.125*		
5	G	T	T	T	G	G	G	C	T	G	A	T	C	G	G	G	T	T	T	G	A	C	C	G	G	A	G	A	T	*0.125*		*0.125*
6	G	T	T	T	G	G	G	C	T	G	G	G	T	G	G	G	T	T	T	G	A	C	C	G	G	—	G	A	T	*0.083*		*0.031*
7	A	T	T	T	G	G	G	C	C	G	A	T	C	G	G	G	T	T	C	G	A	C	C	G	G	—	G	A	T	*0.083*		*0.031*
8	G	T	T	T	G	G	G	C	T	G	G	T	T	G	G	G	T	T	T	G	A	C	C	G	G	A	G	A	T	*0.083*		*0.031*
9	G	T	T	T	G	G	G	C	T	G	G	G	T	G	G	G	T	T	T	G	A	C	C	G	G	A	G	A	T	*0.083*		*0.063*
10	G	T	T	T	G	G	G	C	T	G	G	T	C	G	G	G	T	T	T	G	A	C	C	G	G	A	G	C	C			*0.094*
11	G	T	T	T	G	G	G	C	T	G	G	T	C	G	G	G	T	T	T	G	A	C	C	G	G	A	G	C	T		*0.25*	
12	G	T	T	T	G	G	G	C	T	G	A	G	T	G	G	G	T	T	T	G	A	C	C	G	G	A	G	A	T		*0.10*	
13	G	T	T	T	G	G	G	C	T	G	G	G	C	G	G	G	T	T	C	A	A	C	C	G	G	—	G	A	T		*0.05*	
14	G	T	T	T	G	G	G	C	T	G	A	G	C	G	G	G	T	T	C	A	A	C	C	G	G	—	G	A	T		*0.05*	
15	G	T	T	T	G	G	G	C	T	G	G	T	T	G	G	G	T	T	T	G	C	C	C	C	G	A	C	C	C		*0.05*	
16	G	T	T	T	G	G	G	C	T	G	A	T	C	G	G	G	T	T	T	G	C	C	C	C	G	A	G	A	C		*0.05*	
17	G	A	T	T	G	G	G	C	T	G	A	G	C	G	G	G	T	T	T	G	A	C	C	G	G	A	G	C	C		*0.05*	
18	G	T	T	T	G	G	G	C	T	G	G	T	T	G	G	G	T	T	T	G	C	C	C	C	G	A	C	A	C		*0.05*	
19	G	T	T	T	G	G	G	C	T	G	G	G	C	G	G	G	T	T	T	G	A	C	C	G	G	A	G	A	C		*0.05*	

Only the haplotypes detected with frequency value > 0.05 (>5% population) were considered for analysis. The variant alleles are emphasize in bold. Haplotype number 2 correspond to *ERBB2*_10-15 wild-type sequence (GeneBank: JQ284376). (*) SVs with exonic positions. (*3, 4) Two synonymous sequence variants. (*1, 2, 5, 6, 7) five non-synonymous SVs corresponding to five amino acid changes: Arg46Lys; Val47Glu; Ala205Pro; His206Pro and Val214Ala. (#) g.271T>A and g.271T>G multi-allelic SV; (—) allelic deletion.

Also, from the total SVs observed (30 SVs), the normal samples showed a much lower frequency of variant alleles (12.77%) and heterozygous condition (16.98%) compared to the CMLs (Table S3). In the CMLs the percentage of variant alleles was highest for the benign lesions (18.64%) and similar between the primary malignant and metastatic samples. Moreover, the frequency of the heterozygous condition, was lowest for the MeLs (12.82%) in comparison with the BeLs (20.75%) and MaLs (20.41%) (Table S3).

### Determination of Hardy-Weinberg equilibrium, genotype association and genomic haplotype

With respect to all of the allelic variants detected, 10 loci showed deviations from Hardy–Weinberg equilibrium (p<0.05; Table S3). Basic single allelic and genotypic models of association were used to evaluate the allelic frequency differences between normal and mammary lesions (significant p value <0.05). With the single allelic association test, 5 variants showed significant values of association with the CMLs under analysis: g.355G>A, g.1914G>C, g.2037G>C, g.2041A>C and g.2065T>C. Four of the last 5 also showed significant values of genotypic association with the mammary lesions group: g.1914G>C, g.2037G>C, g.2041A>C and g.2065T>C (Table S3).

The common haplotypes (frequency higher than 5%) were independently determined for three groups of samples: normal samples, CML samples and total samples (Table 1). In general, there was evidence of a strong linkage disequilibrium (LD) between SVs (no random association in the genotypes). The second most frequent haplotype in the normal samples (haplotype 2; Table 1) corresponded to the reference sequence (GenBank: JQ284376). It was also evident that the normal samples showed a distinct haplotype categorisation (haplotype 1–9; Table 1) in comparison with the CML samples (haplotype 10–19; Table 1).

### Probable structural damage effects, homology modelling and molecular dynamics studies of wild-type and variant cat erbB-2 protein

The wild-type *ERBB2*_10/15 (CDS *ERBB2*_10/15) and variant *ERBB2*_10/15 (CDS *ERBB2*_10/15_SV) coding sequences were *in silico* constructed, and the respective reference (erbB-2_10/15) and variant (erbB-2_10/15_SV) protein sequences were subsequently obtained (Figure S2 and Figure S3). A similarity of 98% was found between these two protein sequences. The search for the cat erbB-2_10/15 protein, using the NCBI BLASTP tool, showed a high similarity with cat GenBank: NP_001041628.1 (99%) and with human GenBank: AAO18082.1 (92%) sequences.

The probable damaging effects of the 5 amino acid changes identified in the present work, at the three-dimensional (3D) protein structural level, were evaluated by software analysis using Polyphen, Polyphen-2 and Pymol (Table S4). The five AA changes analysed were: Arg46Lys, Val47Glu, Ala205Pro, His206Pro, and Val214Ala. The software results demonstrated that the Arg46Lys and Ala205Pro changes were benign modifications. However, the remaining three amino acid changes (Val47Glu, His206Pro and Val 214Ala) were classified as probably damaging to the 3D structure of the protein.

**Figure 2:**
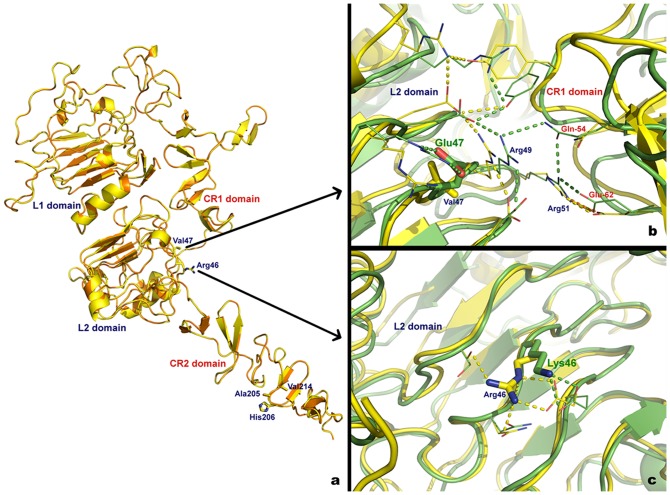
3D models of wild-type and variant cat ErB-2_10-15 protein. (**a**) Superimposed extracellular domain of human erbB-2 (orange) and wt cat erbB-2 homology model (yellow). Residues where mutations were observed are labelled. (**b**) Superimposed conformation of the wt (yellow) and Val47Glu variant (green) cat erbB-2 homology model. (**c**) Superimposed conformation of the wt (yellow) and Arg46Lys variant (green) cat erbB-2 homology model. Structures are represented in cartoon format and H-bonds are represented in traced line using the respective structure colour code.

In order to complete the homology modelling and molecular dynamics studies, a complete wild-type cat erbB-2 homology model was prepared using the human erbB-2 structure as a template. The alignment of both structures revealed a near perfect fit, with a root mean square deviation of 0.073 Å (Figure 2a).

The extracellular domains of the wild-type cat erbB-2 and the 5 variant homology models were then subjected to simulated annealing (SA) tests, and the obtained structures were superimposed onto the initial wt cat erbB-2 homology model. In the SA tests, a considerable rearrangement of the H-bond network with the formation of new H-bonds was detected when comparing wild-type Val47 and variant Glu47 (Figure 2b). Specifically, 2 H-bonds were formed between key residues involved in the CR1/L2 domain interactions: Arg49:Glu54 and Arg51:Glu54 (Figure 2b). Compared to the wild-type protein, the SA simulations performed with the Arg46Lys change showed only minor adjustments observed in the H-bond network and no significant conformational rearrangements were observed in the protein backbone (L2 extracellular domain) (Figure 2c and Table S4).

The SA tests for the remaining 3 variants were not conclusive, as it was impossible to superimpose the wt sequence with the variants. This was probably due to the flexibility observed in the CR2 domain, where the 3 mutations were located (Figure 2).

However, a straightforward comparative analysis between the wt and variant 3D amino acid structures showed that the His206Pro change conferred a polar to non-polar modification in the side chain, and it also changed the residue side chain volume, which could produce a change in the accessible surface propensity of the cat erbB-2_10/15 protein structure. Additionally, the Val214Ala change modified the side chain volume and resulted in the closest contact with the surrounding chains (Table S4)

To improve the comparative analysis between the cat and human protein variants, an extensive search was performed considering all of the non-synonymous polymorphisms described in the dbSNP database (NCBI) for the *ERBB2* cDNA fragment from exon 10–15, corresponding to the cat *ERBB2* reference sequence (GenBank: JQ284376). None of the corresponding AA changes observed in the database had the exact position of the AA changes detected in the present work (data not shown).

### Immunohistochemistry (IHC) erbB-2 protein quantification

For each anti-human erbB-2 antibody used, one specific for the internal domain (CB11/int) and one specific for the external domain (CBE356/ext), the immunoreactivity was assessed according to the Hercep-Test guidelines as described in ASCO recommendations [45]. The two anti-human erbB-2 antibodies used were validated for cat species by western blotting onto cat mammary protein extracts from frozen samples (in RNAlatter solution) (Figure S4). Both antibodies recognized the expected erbB-2 oncoprotein of 185 kDa.

**Figure 3:**
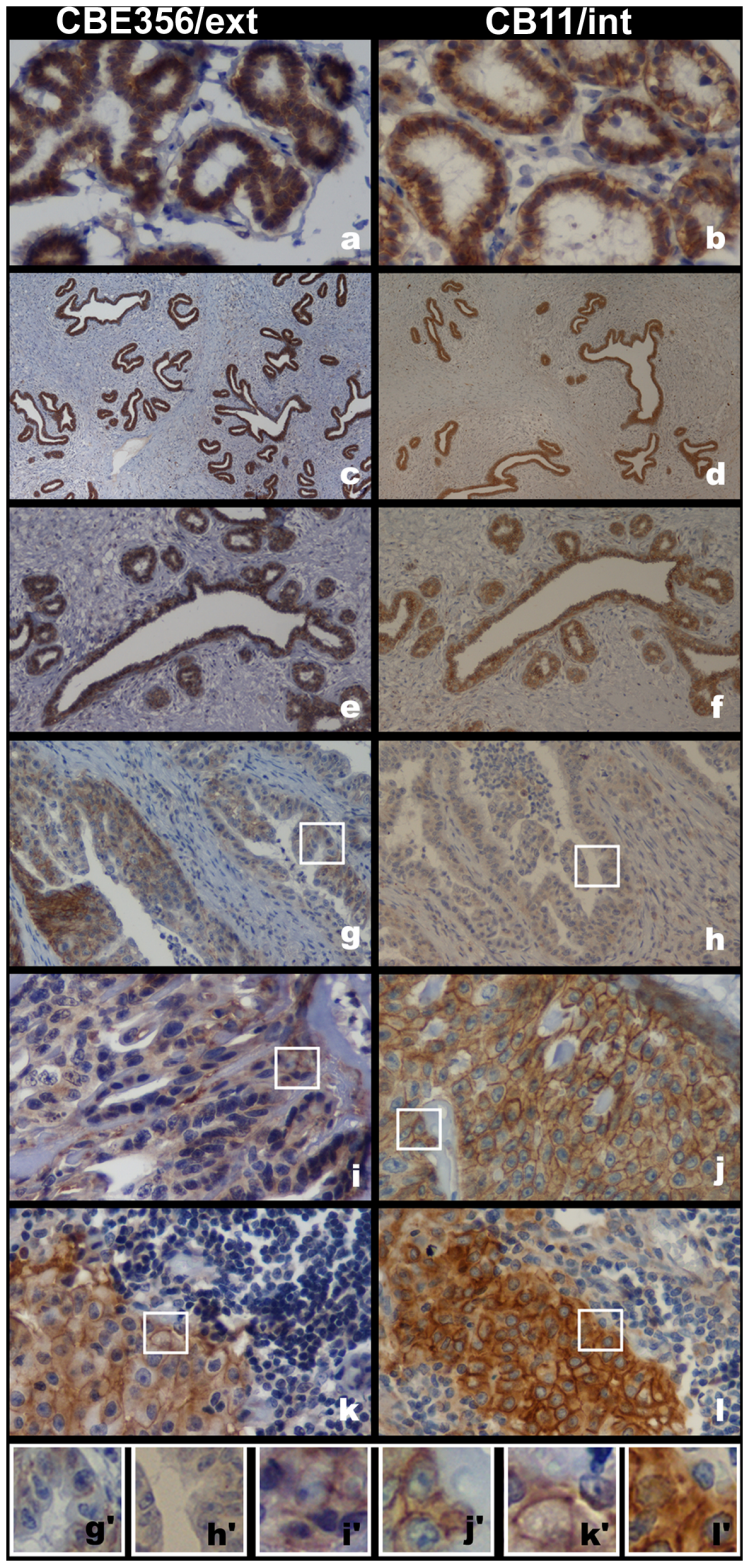
Immunohistochemical evaluation of erbB-2 in cat normal mammary tissues and in mammary gland lesions. Normal samples **(a; b)**, hyperplasic lesions **(c; d)** and neoplastic benign lesions **(e; f)** showing strong and complete membrane labelling (score 3+) with the two antibodies used in IHC test: CB11/int and CBE356/ext. Tubulopapillary lesions depicting 1+ score for HIC CBE356/ext staining **(g)** and no labeling with CB11/int staining **(h)**. Cribiform lesions with moderate and complete membrane labelling (2+) for CBE356/ext antibody **(i)** and 3+ score for CB11/int antibody **(j)**. Lymph node mammary metastatic lesion depicting 3+ scores for both antibodies **(k; l)**. Images original magnifications 400× **(a; b; i-l)**; 40× **(c; d)**; 100× **(e; f)**; 200× **(g; h)**. **(g′-l′)** 300× amplification of the image highlight by white squares in the correspondent **(g-l)**.

**Figure 4:**
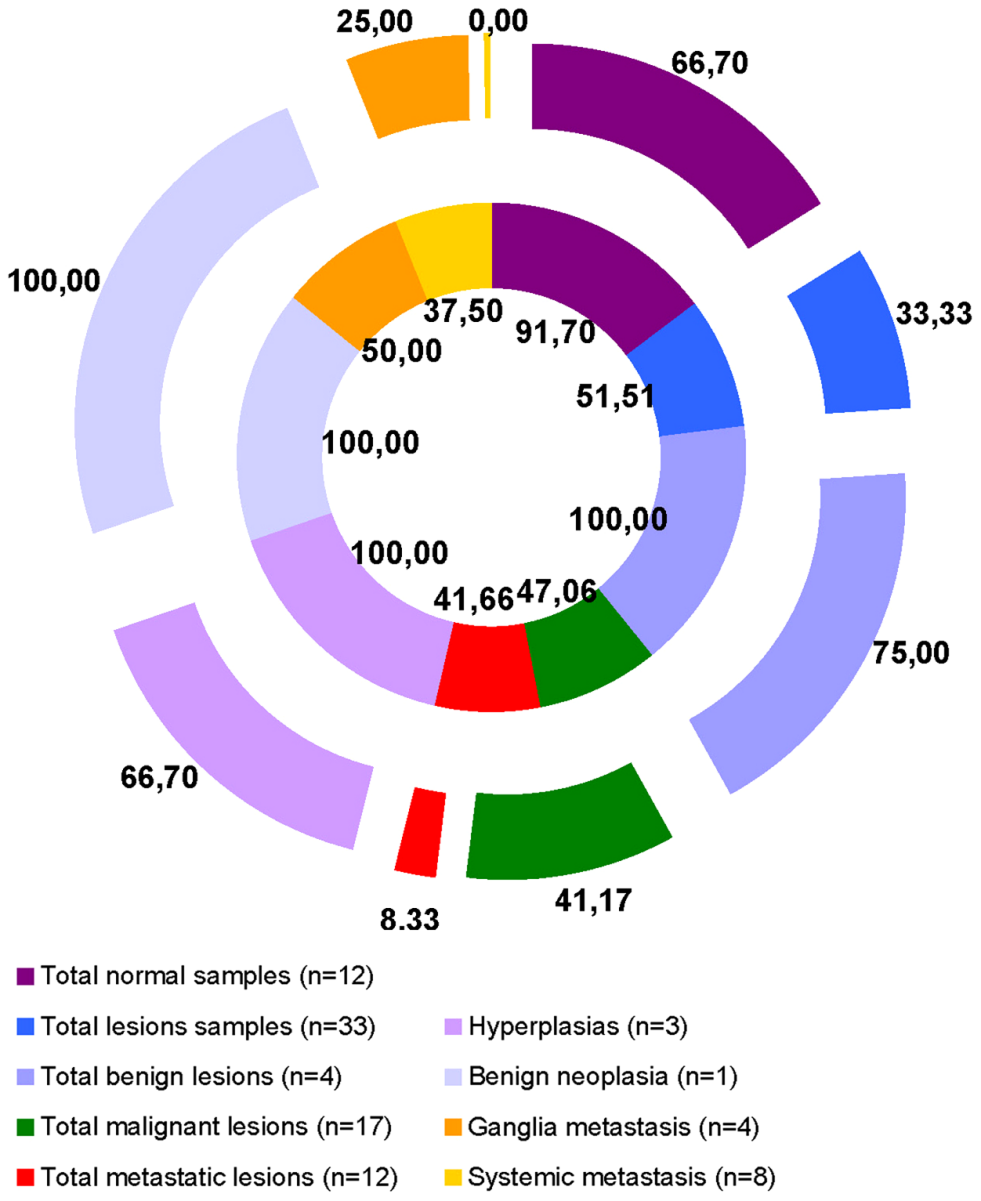
Percentage of immunohistochemistry erbB-2 positives samples. Percentage of HIC erbB-2 positive samples in the different groups of samples. The Results show in outer ring correspond to extracellular antibody (CBE356/ext) and in the inner ring correspond to intracellular antibody (CB11/int).

In accord with the strength of immunostaining, the samples scored as 0 or + were considered negative, whereas those scored as ++ or +++ were considered erbB-2 positives. The IHC scores and erbB-2 negative or positive results described in this subsection are shown in detail in additional table S5, and the IHC results are summarise in figures 3 and 4.

Consistently, the IHC patterns of the erbB-2 protein revealed a disparity in the membrane labelling between the two antibodies. Of the 4 normal mammary tissues studied, 3 were obtained from 2 cats with hyperplasia in a different mammary gland (Table S1: BeL5 and BeL6). Additionally, 8 residual normal mammary glands peripheral to affected mammary tissues (Table S1: MaLs6, 10, 14, 17, 18, 20, 21 and MeL4) were used in the present study. Moderate (erbB-2 ++) or strong (erbB-2 +++) IHC membrane labelling was observed in 66.7% of normal samples for the CBE356/ext antibody and in 91.7% of normal samples for the CB11/int antibody (Figure 3a, b and Figure 4). Also, when we compared the CBE356/ext with CB11/int results, the staining intensities concordance revealed was 50%.

For both antibodies, the CMLs showed a lower percentage of erbB-2 positive tissues than the normal samples. The percentage of positive CMLs was lower for CBE356/ext than for CB11/int labelling (Figure 4 and Table S5). When the IHC results were compared, the scores concordance was higher for the erbB-2 negative (48.48%) than for the positive (27.27%) CMLs samples.

The concordance of the two antibody labelling scores was 50% in the BLs, 23.5% in the MaLs, and 41.7% in the MeLs. The benign lesions revealed a notable incidence of moderate to strong membrane labelling for both antibodies (Figure 3c-f and Figure 4). The primary malignant lesions demonstrated the lowest frequency of erbB-2 positive samples (Figure 3g-j and Figure 4). Of the three lesion types, the MeLs were the least erbB-2 positive. In fact, none of the systemic metastatic samples presented erbB-2 positive results for the CBE356/ext antibody (Figure 4 and Table S5).

Comparing the IHC results from the normal mammary tissues and the matching primary mammary lesions, the staining intensities were concordant in only 9.09% for the CB11/int antibody and 27.27% for the CBE356/ext antibody. The normal samples showed an identical percentage of erbB-2 positive scores compared with the corresponding benign lesions. Relative to the matching primary malignant samples, a markedly higher labelling score was observed in the normal samples for both antibodies. It was only possible to compare the IHC scores of the normal tissue adjacent to 1 mammary lymph node metastasis (Figure 3k, l), which showed high protein scores for both antibodies (erbB-2 positive: MeL +++ and matching normal ++).

In order to verify whether erbB-2 positive is correlated with cat *ERBB2* gene amplification we subjected a total of 20 samples to real time quantitative PCR (qPCR) for gene copy number evaluation. The selection of the 20 samples was as follow: 10 normal and 10 mammary lesions (4, erbB-2 positive; 5, erbB-2 negative; and 1, erbB-2 positive for CBE356 and erbB-2 negative for CB11) (Figure S5). In all cases we didn't detect any gene amplification.

### RT-qPCR *ERBB2* RNA expression analysis

**Figure 5:**
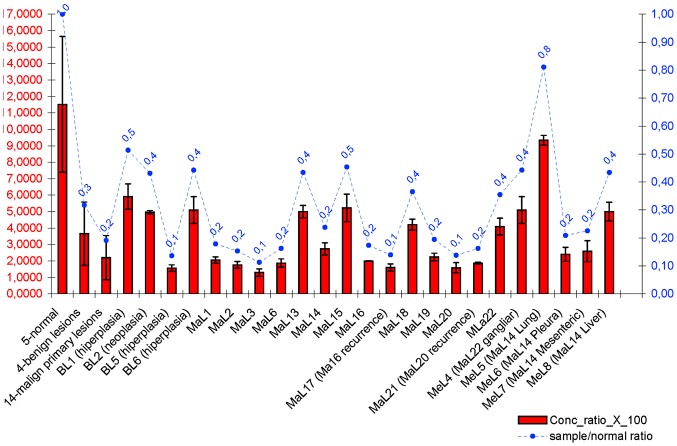
*ERBB2* RNA expression in mammary lesions samples. The graphic with two independent Y axis illustrates the measure of RNA expression output as concentration_ratio_x_100 mean values (left Y axis with red values) with standard deviation values (black lines in red columns) and also the analysis of lesion/normal mean values ratio (right Y axis with blue values) for each sample and groups of samples.

We evaluated the RNA expression status of the *ERBB2* gene in five disease-free mammary samples (normal), four benign lesion samples (3 hyperplasias and 1 neoplastic), fourteen primary malignant lesion samples and five metastatic lesion samples (Figure 5 and Table S1). Our results demonstrated the presence of high *ERBB2* RNA expression rates in the normal mammary samples. All mammary lesion samples showed lower expression levels that the normal samples (Figure 5).

The Student's *t*-test (two-tailed) demonstrated that the disease-free mammary samples showed *ERBB2* RNA expression levels that were significantly higher than the benign (p = 3.4E-09), malignant (p = 6.4E-12) and metastatic (p = 4.07E-12) samples. Also, the benign samples showed significantly higher values of *ERBB2* RNA expression (p = 0.002) compared with the malignant samples (Figure 5).

For comparison of the *ERBB2* RNA levels, we also determined the lesion/normal ratio using the mean values for each individual sample and for the groups of samples (Figure 5). The samples that showed a lesion/normal ratio >0.5 were considered to not be underexpressed (Figure 5: sample MeL5).

We would like to highlight that the MaL16-MaL17 and MaL20-MaL21 samples, corresponding to the first occurrence and recurrence of the mammary lesion, respectively, showed similar expression values: MaL16 (mean ± SD: 2.0000±0.0173)/MaL17 (mean ± SD: 1.6067±0.2193) and MaL20 (mean ± SD: 1.5900±0.3117)/MaL21 (mean ± SD: 1.8733±0.0577) (Figure 5). Additionally, when comparing the primary lesions with the corresponding lymph node lesion (MaL22-MeL4), the RNA expression levels were similar but higher in the metastatic samples. We also observed that different metastatic tissues (MeL5-8) from the same primary malignant lesion (MaL14) showed different expression levels.

### Statistical correlation tests between the clinicopathological features of the mammary lesions, the detected sequence variants, and the IHC and RT-qPCR results

A two-tailed Pearson correlation analysis (confirmed by Spearman's test) was performed, considering all of the clinicopathological features of the mammary lesions, the detected sequence variants, and the mRNA and erbB-2 protein expression levels attained in the present work (SPSS version 17.0; significant p-values >0.05). The significant correlation are summarize in Table 2 (Table S6 and Table S7).

**Table 2:** Resume of the significant two-tailed bivariate correlation results between *ERBB2* RNA, erbB-2 protein expression levels, and clinicopathological features of mammary lesions.

	Protein		RNA		Sequence Variants							
	CB11/Int	CBE356/Ext	Conc ratio X 100	Lesion/normal ratio	g.271 T>G	g.280 G>A	g.311 G>A	g.327 T>G	g.335 C>T	g.1754 A>C	g.1914 G>C	g.1994 del A
**Clinicopathological Features**												
Histological Class	n#*										n*	
Age (Years)							p*					
Lesion size					p*	p*						
Pleomorphism		n*										
Lesions number												n*
Lymphatic invasion								p**		p**	p**	
Vascular infiltration									p*			
Clinical Outcome			n*	n*								
**Protein**					Ni							
CB11/int		p*	p*	p*								
CBE356/ext												
**RNA**					Ni							
Conc_ratio_X_100				p*								

The significant values of the Pearson's correlation analysis were confirmed by the Spearman's test.

(#) Significant correlation only by Spearman's test.

(*) Correlation is significant at the 0.05 level.

(**) Correlation is significant at the 0.01 level.

(p: positive; n: negative) Indication of the direction of the relationship between variables.

(Ni) Sequence variants were not included in correlation test with RNA RT-qPCR and protein IHC.

The correlation test between the sequence variants and the CML clinicopathological features revealed a positive correlation between primary lesion size and high frequency of 2 SVs detected in the normal and CML samples (g.271T>G and g.280G>A). In addition, the SV g.335C>T showed a positive correlation with vascular infiltration, and g.1914G>C showed a negative correlation with the histological classification (Table 2). Regarding the immunohistochemical results, some significant correlations were found. Negative correlations between the CB11/int results and the histological classification and between the CBE356/ext results and nuclear/cellular pleomorphism were observed (Table 2). A positive correlation was observed between the IHC results obtained with the two antibodies (Table 2). Concerning the IHC and RNA expression results, a positive correlation was shown between the IHC results with the CB11/int antibody and the mRNA expression levels. Finally, a positive correlation was discovered between RNA underexpression levels and a worse clinical outcome (Table 2).

## Discussion

### Cat mammary lesions *ERBB2* genomic sequence variant analysis

In the present work, we performed an extensive *ERBB2* gene sequence variant analysis in cat mammary lesions, including neoplastic and non-neoplastic lesions, and a group of normal genomic DNA samples as a control group. Our study focused on the cat *ERBB2* gene from exon 10 to 15, which encodes part of the extracellular domain of the erbB-2 protein. The complete cat *ERBB2* genomic wild-type sequence from exon 10 to 15 (2173 bp) was obtained for the first time in the present work and was submitted to GenBank (accession number JQ284376). Thirty *de novo* SVs were identified in the cat *ERBB2*_10-15 genomic sequence, 6 of which were only observed in the normal samples and 9 of which were only detected in the CML samples. A higher number and frequency of SVs were demonstrated for the CML genomes, and some specific DNA variants were detected in different types of mammary lesions (Table S3).

Seven SVs were localised in exonic regions, 5 of which were revealed to be non-synonymous SVs (nsSVs). Four nsSVs were only detected in the CMLs (JQ284376: g.229T>A, g.2037G>C, g.2041A>C, g.2065T>C). The main outcomes concerning SVs detected in the present study are review in table 3. The 10 SVs detected in normal and CML samples, that showed HWE allelic deviation, may be considered as normal germline variants and could be involved in disease susceptibility (Table S3). A basic single allelic model of association demonstrated that five variant alleles, which were only detected in CMLs, showed significant values of association with mammary lesion samples group (JQ284376: g.355G>A, g.1914G>C, g.2037G>C, g.2041A>C, and g.2065T>C). The determination of the haplotype frequency for cat *ERBB2* using the linkage disequilibrium test uncovered a divergent genotypic haplotype characterisation (nine different frequent haplotypes) between the normal samples and mammary lesion samples (table 1).

**Table 3:** Resume of the main outcomes concerning genomic sequence variants (SVs) detected.

	Genomic sequence variants						
	g.226G>A	g.229T>A	g.355G>A	g.1914G>C	g.2037G>C	g.2041A>C	g.2065T>C
**Minor allele percentage (%)**							
***Normal***	7.69						
***BeL***				37.50	25.00	**37.50**	**25.00**
***MaL***			18.75	25.00	16.67	**33.33**	**25.00**
***MeL***		**16.67**	33.33		16.67	**83.33**	**33.33**
**Heterozygosis percentage (%)**							
***Normal***	15.38						
***BeL***				75.00	50.00	**25.00**	**50.00**
***MaL***			37.50	50.00	33.33	**33.33**	**50.00**
***MeL***		**33.33**	00.00		33.33	**33.33**	**66.67**
**Allelic Association tests**							
***Normal vs. CMLs***	No	**No**	Yes	Yes	Yes	**Yes**	**Yes**
**Genotypic Association tests**							
***Normal vs. CMLs***	No	**No**	No	Yes	Yes	**Yes**	**Yes**
**Clinicopathological** **Correlation**							
***CMLs***	No	**No**	Yes	Yes	No	**No**	**No**
**SVs and AA changes analysis in Total Group of Samples**							
***SVs Position***	Exon 11	**Exon 11**	Intron 12	Intron 14	Exon 15	**Exon 15**	**Exon 15**
***Haplotypes with SVs***	7	**17**	No	15; 16; 18	15; 18	**11; 15; 17**	**15; 16; 17; 18; 19**
***Percentage of SV in Haplotypes (%)***	8	**0.5**	<0.05	0.15	0.1	**0.35**	**0.25**
***AA changes***	Arg46Lys	**Val47Glu**	NA	NA	Ala205Pro	**His206Pro**	**Val214Ala**
***Probably effect in protein***	Benign	**Damage**	NA	NA	Benign	**Damage**	**Damage**

Sequence variants key marks reveal by experimental, statistic and *in silico* analysis. A detailed integrative analysis of nsSVs and their corresponding amino acid changes. The haplotypes corresponds to the frequent haplotypes detected in total samples (frequency >5%). SVs with probably damage effect in protein are in bold text. (%) Percentage. (NA) not applicable. (No) not observed.

With this approach, we intended to contribute new data regarding the role of the *ERBB2* gene in the cat mammary tumour system. As far as we know, this is the first attempt to detect and analyse cat *ERBB2* sequence variants in the genomic fragment from exon 10 to 15 (2173 bp) in normal samples and CML samples. There is only one previous work analysing partial sequences of the cat *ERBB2* gene between exon 17 and 20 (548 bp in length) that demonstrated the presence of two specific SVs and two specific haplotypes in CML samples [44].

The present work demonstrated that the majority of the cat *ERBB2*_10-15 SVs detected were already present in early pre-malignant lesions and that the metastatic lesions showed a lower frequency of heterozygosity. Also, considering only the CML samples, the percentage of the minor allele was higher for the benign samples than in the primary malignant and metastatic samples, demonstrating that some variants were lost during tumour evolution (Table 3 and Table S3). These conclusions corroborated with the previous results, demonstrated for human breast cancer, concerning the idea that progression to an invasive carcinoma is associated with only a few additional changes [46] and that the loss of heterozygosity on a specific genetic variant is associated with genetically advanced tumours [11], [47], [48]. Therefore, our results reiterate that the allelic heterogeneity is found in early human breast lesions and are in agreement with those of various authors who consider the human *ERBB2* proto-oncogene to be a natural target for sequence variant analysis [49], as the encoded transmembrane tyrosine kinase receptor protein is highly recognised to have an important role in human breast cancer prognosis [49]–[51]. Concerning SV studies in human breast cancer, the rs1801200 *ERBB2* polymorphism (detected in exon 17), which corresponds to the Ile655Val mutation in human erbB-2, was extensively investigated as a risk factor for breast cancer by several authors [49], [52]. However, a recent review of case–control studies failed to find any association between the variant allele and breast cancer [53]. This is in agreement with the idea that in a complex system such as the mammary gland cell, a single genetic variant is unlikely to have a strong effect on the clinical phenotype [54]. Some evidence for gene-gene interactions among the sequence variants (SVs-SVs interaction) has been previously demonstrated, supporting the fact that the mechanisms by which single nucleotide polymorphisms influence breast cancer risk is incremental [49], [55]–[58]. Also, it is possible that single genetic variants previously detected, are in linkage disequilibrium with a truly causal allele that remains to be identified [31]. In fact, in the present work 3 of the SVs detected (including 2 nsSVs), were observed in the frequent CML haplotypes, always in the presence of other SVs, that might indicate resistance or susceptibility to breast cancer development.

### Probable structural damaging effects, homology modelling and molecular dynamics studies of wild-type and variant cat erbB-2 protein

To our knowledge, this is the first time that a cat erbB-2 homology model has been published (Figure 2) and that software tests have been performed to investigate the possible effects of amino acid changes on the 3D structure and function of cat erbB-2 (table 3).

All data indicate that the g.226G>A nsSV, only detected in the normal samples, and corresponding to the Arg46Lys benign change, can be considered a normal germline variant.

Comparing the human and cat erbB-2 wild-type models, we demonstrated that the CR1/L2 interaction region showed a conserved network of H-bonds and charged residues. The Val47 is localised in the L2 extracellular domain in the region of the CR1/L2 domain interaction network. In SA simulations, the Val47Glu change, promoted a considerable rearrangement in the hydrogen bond network. The two new H-bonds formed (Figure 2b) are able to further stabilise the CR1/L2 interaction and by exposing the CR1 domain to dimerisation, can help to maintain an active ready form of the cat erbB-2 protein that, as previously demonstrated for human erbB-2 favours the cancer process by promoting signal transduction [59], [60].

The two new H-bonds formed (Figure 2b) are able to further stabilise the CR1/L2 interaction and by exposing the CR1 domain to dimerisation can help to maintain an active ready form of the cat erbB-2 protein that, as previously demonstrated for human erbB-2, favours the cancer process by promoting signal transduction [59], [60].

In addition, the Val47Glu change, classified as possibly damaging to the protein structure, and the corresponding g.229T>A nsSV that was only observed in the metastatic samples should be considered for further analysis, specifically concerning the metastatic process of cat mammary gland carcinomas.

The last 3 amino acid variants (Ala205Pro, His206Pro, and Val214Ala) were detected in the CR2 domain. Our straightforward analysis showed that the His206Pro and Val214Ala amino acid changes altered the side chain polarity and volume, which could modify the accessible surface propensity of the CR2 domain and produce the closest contact with other nearby side chains. Also, the 3 nsSVs, corresponding to these 3 mutations, were identified in the same CML haplotype (haplotype 15; Table 1 and 2). We hypothesise that amino acid variant interactions could have incremental effects on the cat erbB-2 protein structure, function, and therapeutics, as it is known that the CR2 domain plays an important role in human breast cancer treatment. In fact, trastuzumab (Herceptin®) is a monoclonal therapeutic antibody against breast cancer that targets erbB-2 by interacting with the CR2 domain, thus preventing erbB-2 dimerisation (Figure 3a).

In a previous work, Rajasekara and collaborators (2008) studied the effect of nsSVs on the 3D structure of the human erbB-2 protein and its potential use in breast cancer therapy [61]. Moreover, they proposed that Herceptin is the best drug for erbB-2 mutants compared to the native erbB-2 target.

Rajasekara and collaborators (2008) proved that not only do AA changes occur in the CR2 domain, but also changes in the L2 domain could promote conformational alterations in the CR2 domain and consequently change the affinity of the protein to trastuzumab (Herceptin®) [61]. This idea reveals a potential consequence for the Val47Glu AA mutation in the L2 domain in cat erbB-2_10-15 detected in the present work, which was classified as being probably damaging to the 3D erbB-2 structure.

Finally, by comparative analysis, we observed that the 3 AA changes detected in the CR2 domain of cat erbB-2 (Ala205Pro, His206Pro and Val214Ala) do not interact directly with trastuzumab. However, in the human erbB-2 model, residues 566, 567 and 575, which correspond to the Ala205Pro, His206Pro and Val214Ala cat erbB-2 variants, respectively, are localised closer to the major key residues (Glu558, Asp560 and Lys569) [61] and between the loops that mediate the erbB-2 protein-Herceptin interaction (Loop557-561, Loop570-573 and Loop593-603) [55]. This proximity indicates a potential effect on the affinity of the cat erbB-2 variant protein for trastuzumab.

In conclusion, our results concerning sequence variation detection in cat mammary non-neoplastic and neoplastic lesions, are an important contribution to genomic DNA characterisation in cancer, corroborating the idea fomented by Tao and collaborators (2009) for human cancers that large epidemiological studies of predisposed gene polymorphisms can provide new insights into the *in vivo* relationships between genes of interest and cancer risk [49]. In addition to the individual investigation detailed in the present work, the simultaneous occurrence of nsSVs in some haplotypes, that were only detected in cat mammary lesions, could indicate a SV-SV interaction that impacts the effects of the amino acid changes in the 3D structure of the cat erbB-2 protein, which may indicate resistance or susceptibility to breast cancer development and therapy. We believe that further efforts should be made in order to extend the studies of these cat erbB-2 mutations in cat mammary lesions and to analyse their importance in cat mammary tumour evolution.

### Quantification of the erbB-2 protein by immunohistochemistry and *ERBB2* RNA by RT-qPCR

The erbB-2 expression levels in the normal and CML samples were detected by IHC using two antibodies against different regions of the erbB-2 protein: CB11 antibody, which has affinity for the intracellular region (CB11/int); and CBE356 antibody which has affinity for the extracellular region (CBE356/ext). The IHC membrane labelling scores revealed some disparity between the results for the two antibodies and consistently demonstrated superior intracellular expression levels. In the normal samples, we observed a surprisingly higher percentage of moderate to complete erbB-2 membrane labelling (erbB-2 positive) for both antibodies (91.7% CB11/int; 66.7% CBE356/ext). Regarding the cat mammary lesions, the benign lesions generally showed a higher percentage of erbB-2 positivity (100% CB11/int; 75% CBE356/ext), but their values were similar to those of the normal samples. However, the primary malignant (erbB-2 positive: 47.6% CB11/int vs. 41.17% CBE356/ext) and metastatic lesions (erbB-2 positive: 41.66% CB11/int vs. 8.33% CBE356/ext) demonstrated a higher percentage of erbB-2 IHC negative samples (Figure 4). In the different cases tested (normal and mammary lesions) we didn't found any *ERBB2* gene amplification.

In several studies of cat mammary tumours, alterations of the *ERBB2* proto-oncogene have been described, mostly at the protein level [4], [38], [41], [42], [62]. With respect to our IHC erbB-2 analysis, the results for the normal samples are in accordance with the work of Burrai and collaborators (2010), which also found erbB-2 protein expression in normal cat mammary epithelium with strong, complete membrane staining [4]. These findings are contrary to those of other studies that reported no immunoreactivity or a faint, barely perceptible signal in part of the cell membrane in normal cat mammary ducts and acini [41]–[43], [63], and they are also contrary to what has been mainly observed in humans [64]. However, reports of erbB-2 expression in normal human tissues have varied between studies, but it is definite that because of its role in normal cells, the erbB-2 protein is expressed widely in epithelial cells, particularly those of the secretory epithelia, such as the mammary gland [65]. Our data reinforce the idea suggested by Burrai and collaborators (2010) that the erbB-2 protein could be present at higher levels in the normal cat mammary gland compared to the normal human mammary gland [4].

As far as we know, there is only one recent work that also applied antibodies against the intracellular (4B5, A0485 and CB11) and extracellular (TAB250, SP3) regions of the erbB-2 protein, in normal cat glands and cat mammary lesions. However these authors demonstrated that neither TAB250 nor SP3 antibodies showed reactivity with the extracellular regions of the cat erbB-2 protein [62]. The majority of works, with respect to erbB-2 IHC analysis in cat mammary tissues, uses antibodies with affinity for the intracellular region of the erbB-2 protein, which include: A0485 [4], [38], [43]; CB11 [41]; A0485 and CB11 [42]; and, 4B5, A0485 and CB11 [62], [63].

Despite the antibodies used, different authors found dissimilar ranges of erbB-2 protein overexpression (erbB-2 positive IHC) in cat mammary lesions: 76.7–90%, 59.6%, and 39% (2005); 40% (2007); 27% (2010); 5.5–10% (2011), and 20–33% (2013) [4], [38], [41]–[43], [62], [63]. A deeper analysis of the percentage of cat mammary lesions with erbB-2 overexpression obtained in previous works and in the present manuscript demonstrates that with more recent techniques, it appears likely that a low percentage of cat mammary carcinomas overexpress the erbB-2 protein. Thus, it is clear that technical factors can affect the immunohistochemical assessment of erbB-2 expression in cat mammary gland samples [63]. Concordantly, several authors reported that despite the fact that IHC is currently used for human breast cancer erbB-2 protein categorisation, the application of different, common, commercially available erbB-2 antibodies produces variability in the IHC results [65]–[69]. This fact also sustains the recent suggestion that quantification of *ERBB2* mRNA transcripts by RT-qPCR can be used to determine prognoses in breast cancer as an additional molecular test to the erbB-2 IHC test [34]. In human breast cancer, several works refer to *ERBB2* mRNA overexpression in a low percentage of samples [39], [70], and some recent works have also observed low levels of *ERBB2* mRNA, suggesting the occurrence of *ERBB2* mRNA underexpression in HBC [39], [40]. In the single previous publication concerning *ERBB2* RNA expression levels in cat mammary tumours, the authors reported that 55% of the neoplastic lesions showed increased RNA expression [38], but no information was provided regarding the remaining 45%, which suggests that the rest of the samples showed similar or lower RNA expression than the control (RNA pool with 3 normal samples). As discussed for the IHC results, this heterogeneity could be explained by differences in technique, as dissimilar “cut-offs” have been utilised by different authors and many did not quantify the *ERBB2* mRNA expression in normal tissues compared with cancerous tissues [33].

In fact, in the present work, the *ERBB2* RNA expression levels in the cat mammary samples were evaluated by RT-qPCR, applying primers and probes that detected sequences responsible for the translation of the intracellular region of the erbB-2 protein. The results were surprising, as all of the normal mammary samples (n = 5) demonstrated higher expression values than all of the cat mammary lesion samples, which generally corroborated with our IHC results. Also, as all of the benign and primary malignant lesions and 4/5 of the metastatic samples demonstrated a statistically significant *ERBB2* RNA underexpression in relation to the group of normal mammary samples (Figure 3). When comparing the different groups of lesion samples, the primary malignant lesions showed significantly lower expression levels of *ERBB2* mRNA than the benign lesions.

In the present research, we found a positive correlation between the *ERBB2* RNA and protein expression levels in the intracellular region (CB11/int antibody), but a lack of correlation between the *ERBB2* RNA and protein expression levels in the extracellular region (erbB-2 CBE356/ext antibody). These results are in agreement with the fact that *ERBB2* RT-qPCR was performed with primers and probes specific for the RNA sequence that translates part of the intracellular region. This suggests that different transcripts could be involved in the different expression patterns obtained with the two antibodies used, as previously reported in human breast cancers, which also presented a statistically significant correlation between *ERBB2* mRNA RT-qPCR and protein IHC expression levels [71], [72]. In fact, p95, an aberrant form of erbB-2, is not detected by antibodies that target the external domain of erbB-2 because the extracellular domain is missing in this protein [73], [74]. Interestingly, in this work, we found lower levels of CBE356/ext labelling in all tissue sample types, including the normal samples, suggesting the presence of this erbB-2 isoform. Moreover, another mechanism of *ERBB2* gene expression regulation could be involved, such as specific promoter regulation [75], defective RNAs, and mutations in *ERBB2* [76]. Camp and collaborators (2003) speculated that tumours might overexpress another growth factor receptor that promotes tumour aggression via a ligand-dependent or -independent mechanism [39], [76], [77]. Moreover, comparative analysis showed enrichment in alternative events in *ERBB2* over-expressing cells, indicating regulation of alternative splicing mediated by the oncogene [78]. Some authors have illustrated that erbB-2 IHC tests generally reveal a correlation between an erbB-2 positive status and an aggressive phenotype [79], but other authors have proposed that normal levels of the erbB-2 protein could be associated with a similar aggressive phenotype [77], [80]. Additionally, even patients with low erbB-2 expression levels and worse outcome may confer important therapeutic potential being however ineligible for anti-HER2 therapy [80], [81]. This point of view is also in agreement with our results that indicated the presence of some association between low *ERBB2* RNA and erbB-2 protein expression levels with a worse cat mammary lesion prognosis.

## Conclusions

We believe that this work reports the most complete characterisation of the cat *ERBB2* gene in normal samples and cat mammary tumour lesions to date. The present study is the first report to detect amino acid variants in the extracellular region of the cat erbB-2 protein and their potential effects on the interaction of the protein with the therapeutic antibody trastuzumab. The results of our *ERBB2* gene expression analysis suggest the presence of *ERBB2* gene post-transcriptional regulation and the occurrence of proteins with truncations and single point mutations in cat mammary neoplastic lesions. We should emphasise that our IHC and RT-qPCR results assigned the analysed cat mammary primary malignant lesions to a subtype of cat mammary carcinomas that are characterised by *ERBB2* RNA and erbB-2 protein underexpression. Here, we demonstrate the prognostic value of the *ERBB2* gene in cat mammary tumours, identifying this gene into a potential therapeutic target, similar to the results demonstrated for HBC. Additionally, the recurrent occurrence of low erbB-2 expression levels in cat mammary tumours suggests that cat mammary neoplasias could be a valuable model for erbB-2 negative human breast cancer.

## Methods

### Normal and mammary lesion samples

All the owners gave permission to collect the samples of their cats acknowledging that they may be used for research purposes. After owners consent, all samples were collected in accordance with EU Directive 2010/63/EU and approved by the Ethics Committee of Porto University (approval number EC/12-04/POCI/CVT/62940/2004). The samples used in the presented work have been describe in previous publications [44], [82]. All cat mammary lesion samples were histologically classified according to the diagnostic criteria proposed by the World Health Organization (WHO) classification of mammary tumours of the dog and cat [83]. When available, clinical characteristics, including age at diagnosis, previous diseases and number of lesions, were obtained from medical records over a period of 2 years [84]. A mammary lesions histological and clinical grading evaluation (including prognostic factors) was execute accordingly with Gimenez and collaborators [85] and Misdorp and collaborators [6].

For the sequence variant analysis, we used 14 blood samples and 15 fresh mammary lesion samples. In addition, we used 4 cat mammary carcinoma formalin-fixed paraffin-embedded tissues (FFPET) from an archive. The fresh mammary samples were recovered during mastectomy and immediately frozen in order to preserve the DNA. Ten normal samples (blood) were obtained from animals that also bore a mammary gland lesion.

To measure the *ERBB2* RNA expression levels, we collected 5 normal mammary glands and 23 fresh mammary lesion samples. These samples were frozen (−80°C) in a RNA stabilisation solution (RNA Later Tissue Collection, Ambion). The normal mammary gland samples were obtained from five different queens at necropsy with no evidence of pathological mammary conditions. The mammary lesion samples were collected during mastectomy.

The immunohistochemical (IHC) erbB-2 protein quantification analyses of the mammary lesion samples were performed on the samples used for the quantification of RNA expression (except 1 malignant lesion and 1 metastatic sample were unavailable for this procedure). Additionally, 7 metastatic lesions and the 4 FFPET samples utilised in the genomic SV study were also used in the IHC analyses. A total of 21 primary and 12 metastatic CML samples were submitted to IHC. Additionally, 12 normal mammary tissues were analysed (4 normal mammary samples and 8 residual normal mammary glands peripheral to the mammary lesions).

### Extraction of genomic DNA and total RNA

The nucleic acids from the FFPET (genomic DNA - gDNA) and fresh/frozen samples (gDNA and RNA) were extracted and the quality and concentration were evaluated as described previously [82], [86].

### Analysis of the cat *ERBB2*_10/15 fragment in normal samples

The four primers used were designed based on two cat sequences from the National Center for Biotechnology Information (NCBI) genome browser (cat *ERBB2* mRNA, GenBank:AY702651.1; cat *ERBB2* DNA, GenBank:AY685128) and the human sequence (human *ERBB2* DNA, GenBank:NG_007503). The primer sequences were as follows: E10 sense, 5′-GGACCCAGCCTCCAACACTG-3′; E12 sense, 5′-GGACGAGTGCGGTAAGACAG-3′; E14 antisense, 5′-AGGTCACTGAGCCATTCTGG-3′ and E15 antisense, 5′-GAGTGGGTGCAGTTGATGGG-3′. Polymerase chain reaction (PCR) experiments and PCR products evaluations were performed according to Santos and collaborators [44]. In order to amplify and sequence the *ERBB2*_10-15 gene fragment, the primer set E10/E15 was applied using gDNA obtained from two blood samples from two cats with no history of mammary disease. The PCR fragments were excised from the agarose gel, purified (Geneclean II kit; BioGene), and cloned into the pCR 2.1-TOPO® vector (TOPO TA cloning® kit; Invitrogen/Life Technologies). The plasmid DNA was extracted (Quickgene DNA plasmid kit; Fujifilm/Life Sciences), and four positive clones were selected for sequencing. The sequences were evaluated with Vector NTI software (Invitrogen/Life Technologies). The sequences were determined in both directions using the DNA Sequencing Kit (ABI Prism).

### *ERBB2*_10-15 sequence variants detection in normal and mammary lesion samples

To perform gDNA sequence variant detection in normal and CML samples, gDNA fragments were amplified with two sets of primers: E10/E14 (from exon 10 to 14) and E12/E15 (from exon 12 to 15). The positive PCR reaction products were purified, and sequencing was performed in both directions. The sequences were edited directly with the chromatograms in the ContigExpress Vector NTI module (Invitrogen/Life Technologies), and a final consensus sequence was established for each sample.

### *ERBB2*_10-15 coding sequence prediction and *in silico* analysis

A search for human and cat *ERBB2* exon structure was performed using the NCBI, Ensembl and GeneCards browser databases. A comparative analysis allowed for the recognition of the boundaries of exon 10 to 15 in the genomic *ERBB2*_10-15 wild-type (wt) sequence. We constructed a variant genomic sequence (*ERBB2*_10-15_SV) *in silico* that comprised all of the detected variant alleles (Vector NTI 10.3.0 software; Invitrogen/Life Technologies). Coding region sequences (CDSs) and protein sequences were determined based on the wild-type and variant DNA sequences. This intricate analysis involved the validation of the gDNA, DNA coding region and protein sequences obtained from the genome and protein databases (Ensembl, UCSC Genome Browser, UniProtKB/TrEMBL and NCBI) by alignment.

### Determination of Hardy-Weinberg equilibrium, genotype association and genomic haplotype

All statistical tests described in this subsection were performed with SVS7 software (SNP and Variation Suite™ 7; Golden Helix). Chi-square test results were considered to have significant values at 5% (p-value <0.05). Changes in the HWE were evaluated and verified by chi-square tests for all cat *ERBB2* SVs detected in our experiment. Basic single allelic and genotypic models of association (chi-square test) were used to evaluate the allelic and genotypic frequency differences between the normal and mammary lesion samples. SVs with a p-value <0.05 were considered to be associated with cat mammary lesions.

The haplotype frequencies were estimated for the three groups of samples: normal samples, mammary lesion samples and total samples. The haplotype determination was based on the pairwise measure of the linkage disequilibrium between all of the SVs detected (D′ statistic) [87]. The chi-square test was used to test associations between the SVs in order to identify haplotypes.

### Computational analysis, homology modelling and molecular dynamics studies for the cat erbB-2 protein

PolyPhen and PolyPhen-2 software was used to identify homologues via a BLAST search of the nrdb database (Natural Resources Database) and a BLASTP query sequence against the protein structure database (PDB: Protein Data Bank). The resulting multiple alignment was used to compute the absolute PSIC (Position-Specific Independent Counts) value of the difference between the profile scores of both allelic variants in the polymorphic position. PolyPhen uses empirically-derived rules to predict that an nsSV is probably damaging, possibly damaging, or benign to protein function or structure. The output shows several characterisations of the substitution site, such as a hydrophobicity change and changes in the residue side chain volume (measured in Å^3^). In addition, the automatic bioinformatics tools of PolyPhen-2 predict whether an amino acid substitution affects the protein function or stability based on sequence homology and the physical properties of amino acids mapped in a substitution site of the three-dimensional (3D) structure of a known protein. The output includes a probability score that classifies the mutation as possibly damaging (score <0.15), probably damaging (score > 0.85) or benign (score 0.15–0.85).

In order to complete the homology modelling and molecular dynamics studies, a cat erbB-2 wild-type homology model was built using the SWISS-MODEL online homology modelling server [88]. The complete human erbB-2 structure was kindly supplied by Dr. Péter Bagossi [89] and was used as a template file for the SWISS-MODEL server. From the prepared wt cat erbB-2 model, different homology models of the 5 detected variants were established by incorporating the respective mutation. Using the Protein Preparation Wizard protocol available in the Maestro software program [90], the extracellular domains of the homology models of cat *ERBB2* wt and the 5 variants were prepared, with hydrogens added and water molecules removed. The wt and variant 3D models were aligned using Pymol software, and a preliminary comparative analysis was performed.

Before applying the simulated annealing (SA) protocol, Desmond software (D.E. Shaw Research, New York, NY) was used to add water molecules around the proteins and sodium ions to neutralise the negative charges. All structures were then subjected to SA. The stages used in this study were standard for SA simulations using Desmond software. The simulation time was 1.2 ns with a recording interval of 1.2 ps. The system was heated from 0 to 400 K and then stabilised at 300 K and 1 atm using the Berendsen thermostat in six separate stages. The last frame of each simulation was used to structurally analyse the variants. Structural alignments and image preparations were obtained using Pymol software.

### Protein quantification by immunohistochemistry

Tissues were fixed in 10% buffered formalin (≤48 hours), and embedded in paraffin. Three consecutive, 3 µm thick sections were cut; one was stained with haematoxylin and eosin for histopathologic diagnosis, and the others were used for the IHC study. The haematoxylin/eosin-stained sections were independently examined by two pathologists.

For the IHC study, sections were deparaffinised and hydrated, and antigen retrieval was performed in a pressure cooker in 10 mmol/L sodium citrate buffer (pH 6.0) for 2 min. The slides were cooled for 10 min at room temperature and rinsed twice in triphosphate buffered saline (TBS) for 5 min. After blocking endogenous peroxidase with 3% hydrogen peroxide in methanol for 10 min, the sections were incubated with the following two monoclonal antibodies at a dilution of 1∶40 mouse anti-human erbB-2 oncoprotein CB11, which is specific for the internal domain (CB11/int), and mouse anti-human erbB-2 oncoprotein CBE356, which is specific for the external domain (CBE356/ext) (Novocastra, Newcastle, UK). The sections were subjected to immunohistochemical staining for 90 min and visualised with the Novolink™ Max-Polymer detection system (Novocastra). The sections were rinsed with TBS between each step of the procedure. Colour was developed for up to 7 min at room temperature with a freshly prepared solution of 3,3′-diamino-benzidine, and the sections were then lightly counterstained with haematoxylin, dehydrated and mounted. ErbB-2 positive human breast carcinoma samples were used as positive controls, while the primary antibody was replaced by isotype-specific IgG in negative controls.

The immunoreactivity of erbB-2 was assessed according to the Hercep-Test scoring criteria, described in the American Society of Clinical Oncology (ASCO) guidelines [45]. A minimum of 1,000 cells were counted in at least 10 random high-power (400×) fields. In accord with the strength of immunostaining, the samples were scored as: 0, no labelling; + (or 1+), weak and incomplete membrane labelling; ++ (or 2+), weak to moderate complete membrane labelling of at least 10% of the tumour cells; and +++ (or 3+), strong and complete membrane labelling of at least 10% of the tumour cells. Samples showing areas with different scores (0/+, +/++ or ++/+++), were evaluated in agreement with erbB-2 scores in 10% of tumour cells in the total area analysed. Cat mammary lesions scored as 0 or + were considered erbB-2 negative, whereas those scored as ++ or +++ were considered erbB-2 positive for immunostaining.

### SDS-PAGE and Western Immunoblotting

Protein was extracted from frozen samples at −80°C preserved in RNAlater (Ambion, Invitrogen Life Technologies), of 2 cat mammary lesions and 2 normal mammary glands. Protein extraction was performed with Qproteome Mammalian Protein Prep Kit (Qiagen) in accordance with manufacturer's recommendation. After extraction the protein were stored at −20°C until needed. For electrophoresis, before being loaded, samples were prepared by mixing the protein extract (3 µl) with 5xSDS loading buffer (12 µl) and denaturing at 95°C for 5 min. The proteins were loaded on a NuPAGE® Novex 4–12% Bis-Tris Mini Gel (Invitrogen Life Technologies) and subjected electrophoresis with MOPS running buffer at a constant voltage of 200 V for 50 min (XCell SureLock™ mini-cell electrophoresis system; Life technologies). BlueStar Plus Prestained protein marker (Nippon Genetics Europe) was included in the gel. The proteins were stained with AdvanStain Scarlet (Advansta) and digitized with an imager system (UV).

For western immunoblots, electrophoresed proteins were transferred to PVDF membranes in the iBlot® gel transfer device (iBlot® gel transfer stacks; Invitrogen Life technologies), and blocked with 5% skim milk in PBS-T (phosphate buffered saline with 0.05% Tween 20) for 1 hour. The antibodies were prepared in PBS-T with 2% skim milk. Half of the membrane was then incubated with the anti-human erbB-2 oncoprotein CB11 antibody at 1: 300 dilution. The other half of the membrane was incubated with the anti-human erbB-2 oncoprotein CBE356 antibody at 1∶80 dilution (Novocastra, Newcastle, UK) for 1 h or overnight at 4°C with agitation. The membranes were then washed with PBS-T with 1% skim milk, and incubated for 1 h with, HRP conjugate goat-anti-mouse secondary antibody (Advansta) in PBS-T plus 2% skim milk. The results were visualized with chemiluminescent HRP substrate (WesternBright™ ECL kit; Advansta).

### *ERBB2* DNA copy number absolute quantification

For *ERBB2* absolute quantification we used the standard curve method. The PCR primers for *ERBB2* analysis were: 5′-GAGTGCGGTAAGACAGGGAG-3′ and 5′-AGTCTGCACAAGTCCGAGAT-3′. A 2-fold serial dilution series of the plasmid DNA with the *ERBB2* insert already referenced in this section (cf. “Analysis of the cat *ERBB2*_10/15 fragment in normal samples”), ranging from 3.12×10^7^ to 1.95×10^6^ copies, was used to construct the standard curve (5 points series dilutions) (standard curve in supplementary material; Figure S5). The concentration of the plasmid with the insert was measured using the NanoDrop ND-1000 (NanoDrop Technologies) equipment and the corresponding plasmid copy number was calculated

using the following equation: DNA (copy number) = [6.023×10^23^ (copy number/mol) × DNA amount (g)]/[DNA length (bp) ×660 (g/mol/bp)], where Avogadro number is 6.023×10^23^ (copy number)/1mol and the average molecular weight of a double-stranded DNA molecule is 660 g/mol/bp. In the respective formula the recombinant plasmid DNA length is 4650 bp (pCR 2.1-TOPO vector 3900 bp and the insert 750 bp).

*C*_T_ values in each dilution were measured using real-time qPCR to generate the standard curve for *ERBB2*. Briefly, the standard curve includes a plot of the *C*_T_ values versus the log concentration of the *ERBB2* standard. For normal and tumour lesions cat genomic DNA, the unknown total DNA sample, was obtained by interpolating its *C*_T_ value against the standard curve. We analysed 10 normal cat mammary samples and 12 cat mammary lesions (supplementary material, Figure S5). In the PCR reaction we used 20 ng of genomic DNA. The reactions were performed with the MeltDoctor HRM Master Mix, which use the SYTO9 dye (Applied Biosystems, Invitrogen Life Technologies) following the recommendations of the manufacture. This experiment was carried out in the StepOne real-time PCR system (Applied Biosystems, Invitrogen Life Technologies), where the samples were subjected to an initial denaturation at 95°C (10 min), and then to 40 cycles at 95°C 15 sec followed by 61°C 1 min. Afterwards a melt curve was performed in order to evaluate the primer specificity. All reactions were performed in triplicate, and negative controls (without DNA) were also run.

The StepOne software (version 2.2.2, Life Technologies Applied Biosystems) was used to generate the standard curve and for data analysis. Only standard curves with the following parameters were considered to be typically acceptable: R^2^>0.99 and slopes between −3.1 and −3.6 giving reaction efficiencies between 90 and 110%.

The absolute quantification of *ERBB2* allowed determining the copy number of this sequence in cat mammary normal and lesions genomes that corresponds to 20 ng which, in turn, comprises 5714 haploid genomes. The mass of *Felis catus* haploid genome was obtained in Genome Size database (http://www.genomesize.com/) as being 3.5 pg (3.5×10^−3^ ng).

### RNA expression analysis by quantitative real-time polymerase chain reaction (RT-qPCR)

Primers were designed and probes were selected against conserved cat and human cDNA sequences corresponding to the *ERBB2* sequence that encodes the internal region of the cat erbB-2 protein. Primers (F, 5′-CTTTTGGGGCCAAACCTTAT-3′ and R, 5′-CTAGTGGGACGCGGACAT-3′) were designed using Roche LightCycler Probe Design Software (version 2.0). The concentration of the working solution was 20 µM for all oligomers. The hybridisation probe was selected from the Human Universal Probe Library from Roche Applied Science (UPL58). The *GAPDH* gene was used as a *housekeeping* gene. The qRT-PCR analysis was performed according to the methodology previously described by Baptista and collaborators [85].

The Student's *t*-test (two-tailed) was performed using Microsoft Excel 2007. Statistical significance was defined as p<0.05.

### Statistical correlation tests between the clinicopathological features of the cat mammary lesions, detected sequence variants, and IHC and qRT-PCR results

For this analysis, we calculated two values: Pearson's correlation coefficient (measure of linear association) and Spearman's rho coefficient (quantitative variables or variables with ordered categories). Correlations were considered to be significant at p-values of 0.05 (5% significance) and 0.01 (1% significance), respectively. Correlation coefficients obtained with less than 5 match values were not considered in the final analysis. The data was analysed assuming that, for each clinicopathological characteristic, a higher value corresponded to a worse prognostic/clinical evaluation (Table S2). We completed our study with a bivariate correlation analysis between the different clinicopathological features of the cat mammary lesions with RNA expression levels and immunohistochemical scores (Table S6). The correlation test with the SVs detected, was performed only with the clinicopathological features (Table S7).

## Supporting Information

Figure S1 ***ERBB2*** **DNA partial sequence corresponding to exons 10–15 align study.** Multi-alignment between reference (Cat *ERBB2* DNA 10–15) and variant (Cat *ERBB2* DNA 10–15 variant) cat *ERBB2* DNA sequences and the corresponding Human *ERBB2* variant 2 mRNA sequence (Human *ERBB2* mRNA 10–15). The genomic SVs are black highlight. (N) Multi-allelic SV present in genomic position 271 (g.271 T>G and g.271 T>A). **Adobe (.PDF), Paper size 18×45 cm.**
(DOC)

Figure S2 **Align study of** ***ERBB2*** **partial coding sequence corresponding to exons 10–15 transcripts.** Multi-alignment between wild-type sequence (Cat *ERBB2* CDS 10–15 wt) and variant (Cat *ERBB2* CDS 10–15 variant) cat *ERBB2* cDNA sequence and the corresponding Human *ERBB2* variant 2 mRNA sequence (Human *ERBB2* mRNA 10–15). The cat *ERBB2* CDS sequences were obtained by introns sequences deletion from the reference and variant sequences (submitted to GeneBank). The nsSVs detected in CDS are in red. The synonymous SVs detected in CDS are in green. **Adobe (.PDF); paper size A4.**
(DOC)

Figure S3 **Align study of ErbB-2 partial protein sequence corresponding to exons 10–15 translation.** Multi-alignment between wild-type (Cat erbB-2 10–15 protein wt) and variant cat erbB-2 protein sequence and with the corresponding Human erbB-2 partial protein sequence. The Human sequence correspond to part of the chain A from the 3BE1 model. The amino-acid changes detected are indicated in red. **Adobe (.PDF); paper size 21×15 cm.**
(DOC)

Figure S4 **Western blot with the CBE356 and CB11 anti-erbB2 antibodies.** Reactivity observed by western immunoblot with the CBE356 and CB11 anti-erbB2 antibodies, in 3 feline mammary tissues. Lane 1: non-neoplastic cat mammary tissues; Lanes 2-3: cat mammary lesion samples. The expected molecular weight (185 kDa) of the reactive protein band, was determined by comparison, with the molecular weights of the standard Protein Marker (PM). **Adobe (.PDF); paper size 21×15 cm.**
(PDF)

Figure S5 **ERBB2 DNA copy number quantification: standard curve construct and copy number/haploid genome assessment.** (A) Standard curve constructed with a 2-fold serial dilution series of the plasmid DNA with the *ERBB2* insert, ranging from 3.12×10^7^ to 1.95×10^6^ copies (5 points series dilutions, each point in triplicate). The Efficiency of the PCR reaction was 98% (R2 = 0.996). (B) 10 normal and 10 mammary lesions were subjected to real time quantitative PCR for gene copy number evaluation. In all cases we didn't detect any gene amplification. (IHC) Immunohistochemistry. **Adobe (.PDF); paper size 21×15 cm.**
(PDF)

Table S1 **Summary of the clinicopathological evaluation and clinical outcome of the cat mammary benign, malign and metastatic lesions study in the present work.** Tumour identification (ID) in 3 categories: (BL) benign lesions; (MaL) primary malign lesion; (MeL) metastasis lesion. (neo) neoplastic; (hip) hyperplasia; (LN) Lynph node; (rec) recurrence; (NE) No evidence; (NA/E) Not applicable/evaluated. Clinical outcome evaluation: (DOD) dead of disease; (DC) developed a carcinoma (NED): no evidence of the disease; (*NED) no evidence of the disease for more than 2 years; (DOC) dead of other causes. (#) Histological classification in accord with Misdorp *et al*. (1999) [83]. The evaluation grade is in accord with Gimenez *et al*. (2010) [85] and Misdorp W. (2002) [6] (describe in Table S2). **Word (.doc); Page size 33×33 cm.**
(DOC)

Table S2 **Cat mammary lesions clinical and histological grading evaluation features and other prognostic factors.** Accordingly with Gimenez *et al*. (2010) (*) [85] and Misdorp, W. (2002) (#) [6]. The scores grading presuppose that, for each clinicopathological characteristic, a higher value corresponded to a worse prognostic/clinical evaluation. **Word (.doc); Page size A4.**
(DOC)

Table S3 **Analysis of sequence variants detected in the Cat** ***ERBB2*** **gene fragment between exons 10 to 15.** A total of 30 SVs were detected with dissimilar observation of 15 SVs in normal and CML samples. The minor allele and the genotype heterozygosis frequencies (%) were calculated individually for each SV. The Hardy-Weinberg equilibrium is indicated by the frequency value (p-value) calculated by the Chi-square test. The SVs that present p<0.05 are considered to present HWE deviation. The probable amino-acid changes are indicated whit the respective number of the amino-acid in the cat erbB-2_10/15 protein sequence. Two synonymous (Syn.) and five non-Synonymous (Non-Syn.) variations were detected in exonic positions. The zero percentage values were removed for better visualization. **Footnotes:** (*) 6 SVs only in normal samples; (#) 9 SVs only in mammary lesions samples; (a) SVs with significant values of allelic association with the CMLs; (g) SVs with significant values of genotypic association with the CMLs. **Word (.doc); paper size: 29×25 cm.**
(DOC)

Table S4 **Resume of the AA change effects in cat erbB-2 protein: results of the analysis in Pymol and Polyphen/Polyphen-2 software's.** (nc) no changes detected. The categorization results obtained with Polyphen and Polyphen-2 bioinformatics automatic tools are the same. **Word (.doc); paper size A4.**
(DOC)

Table S5 **Cat erbB-2 immunohistochemical staining results for each antibody used: CBE356, specific for the external domain and CB11, specific for the internal domain.** The strength of immunostaining was estimated as negative (0 and +) or positive (++ and +++). (*) 3 normal mammary samples from 2 hyperplasia cases; (a) Tubulopapillary carcinomas with solid, mucinous or different disease stage; (b) Systemic metastasis; (neo-BL) neoplastic benign lesions; (MaLs) primary malign lesions. **Word (.doc); paper size: 25/40 cm.**
(DOCX)

Table S6 **Correlation measure between** ***ERBB2*** **RNA and erbB-2 protein expression levels and clinicopathological features of primary mammary lesions.** (Red *) Correlation is significant at the 0.05 level. (Red **) Correlation is significant at the 0.01 level. (Corr.) Correlation tests Output. (N) Number of rows in working input data file. (-) Negative relationship between variables. Two-tailed bivariate correlation analysis calculated with indication of two significance levels (p-value not showed). The correlation significant values of the Pearson's analysis were confirmed by the Spearman's test. **Word (.doc); paper size A4.**
(DOC)

Table S7 **Correlation measure between cat** ***ERBB2*** **_10–15 Sequence Variants detected and clinicopathological features of cat mammary lesions.** Two-tailed bivariate correlation analysis calculated with indication of two significance levels (p-value not showed). The significant values of the Pearson's correlation analysis were confirmed by the Spearman's test. (*) Correlation is significant at the 0.05 level. (**) Correlation is significant at the 0.01 level. (-) negative correlation between the variables. (a) Cannot be computed because at least one of the variables is constant. **Word (.doc); paper size 40×20 cm.**
(DOC)
