# Supplementary material for: ERBB2 in Cat Mammary Neoplasias Disclosed a Positive Correlation between RNA and Protein Low Expression Levels: A Model for erbB-2 Negative Human Breast Cancer
Source: PLoS One. 2013 Dec 26;8(12):e83673. doi: 10.1371/journal.pone.0083673 (PMC3873372; doi:10.1371/journal.pone.0083673)
Supplement: Figure S1 — ERBB2 DNA partial sequence corresponding to exons 10–15 align study. Multi-alignment between reference (Cat ERBB2 DNA 10–15) and variant (Cat ERBB2 DNA 10–15 variant) cat ERBB2 DNA sequences and the corresponding Human ERBB2 variant 2 mRNA sequence (Human ERBB2 mRNA 10–15). The genomic SVs are black highlight. (N) Multi-allelic SV present in genomic position 271 (g.271 T>G and g.271 T>A). Adobe (.PDF), Paper size 18×45 cm. (DOC) [file pone.0083673.s001.doc]

**Additional Figures; Santos *et al*.; Adobe (.PDF), Paper size 18x45 cm**

**Figure S1: *ERBB2* DNA partial sequence corresponding to exons 10-15 align study.**

Cat ERBB2 DNA 10-15 (1) GGACCCAGCCTCCAACACTGCCCCCCTGCAGCCTGAGCAGCTCAGAGTGTTTGAGGCTCTGGAGG

Cat ERBB2 DNA 10-15 Variant (1) GGACCCAGCCTCCAACACTGCCCCCCTGCAGCCTGAGCAGCTCAGAGTGTTTGAGGCTCTGGAGG

HSA ERBB2 DNA 10-15 (1) GGACCCAGCCTCCAACACTGCCCCGCTCCAGCCAGAGCAGCTCCAAGTGTTTGAGACTCTGGAAG

Cat ERBB2 DNA 10-15 (66) AGATTACAGGTGGGCTTTGCTTCTCTGCATCCTGCTCTGATGGGGTCGGGGGTCCTCTTCCTGTC

Cat ERBB2 DNA 10-15 Variant (66) AGATTACAGGTGGGCTTTGCTTCTCTGCATCCTGCTCTGATGGGGTCGGGGGTCCTCTTCCTGTC

HSA ERBB2 DNA 10-15 (66) AGATCACAGGTGGGCTCTG--TCTCTGCATCCTGTTCTGCAGGGGCTGGGAGTCCTTGTCCTGTC

Cat ERBB2 DNA 10-15 (131) CCCACACCCCTAGCCTCACCCTGTGCCCGCAGGTTACCTGTACATCTCAGCGTGGCCAGACAGCT

Cat ERBB2 DNA 10-15 Variant (131) CCCACACCCCTAGCCTCACCCTGTGCCCGCAGGTTACCTGTACATCTCAGCGTGGCCAGACAGCT

HSA ERBB2 DNA 10-15 (129) CCCACTCCTTTAATCTCACCCTCTGCCTGCAGGTTACCTATACATCTCAGCATGGCCGGACAGCC

Cat ERBB2 DNA 10-15 (196) TGCCTAACCTCAGTGTCTTCCAGAACCTCA**G**AG**T**GATCCGGGGCCGAGTTCTGCATGAGTGAGCA

Cat ERBB2 DNA 10-15 Variant (196) TGCCTAACCTCAGTGTCTTCCAGAACCTCA**A**AG**A**GATCCGGGGCCGAGTTCTGCATGAGTGAGCA

HSA ERBB2 DNA 10-15 (194) TGCCTGACCTCAGCGTCTTCCAGAACCTGCAAGTAATCCGGGGACGAATTCTGCACAAGTGAGCA

Cat ERBB2 DNA 10-15 (261) CCGGGGAAG**TT**GG**G**GGGGGG**G**C**CT**GAGGACCTTCCCAG**G**GAG----CCCGTTGA**G**AAGCTCTGGG

Cat ERBB2 DNA 10-15 Variant (261) CCGGGGAAG**GN**GG**T**GGGGGA**C**C**-C**GAGGACCTTCCCAG**A**GAG----CCCGTTGA**A**AAGCTCTGGG

HSA ERBB2 DNA 10-15 (259) CTGAGAAAGAGGGGGCCTGATGGGGAGGAG--TCCCAGGGAGGAGTCCCTGTGGGAAGCTTTGGG

Cat ERBB2 DNA 10-15 (322) CATGG**T**GGGCT-CT**C**CTGTTCCCCAGG**G**ACTCCA**G**CAGTAACATTTCCATGGGAGTTTTCGGAGT

Cat ERBB2 DNA 10-15 Variant (322) CATGG**G**GGGCT-CT**T**CTGTTCCCCAGG**A**ACTCCA**A**CAGTAACATTTCCATGGGAGTTTTCGGAGT

HSA ERBB2 DNA 10-15 (322) CCTGAGGGAGTACTCCTGT--------------AGCAGTAACCTTTCCATGAAAGTCTGCAGAGT

Cat ERBB2 DNA 10-15 (386) GTGCTGGGGACAGGAGGAGGTGAGGGCAGCTCTTGCCGACAGCCAA-------------------

Cat ERBB2 DNA 10-15 Variant (386) GTGCTGGGGACAGGAGGAGGTGAGGGCAGCTCTTGCCGACAGCCAA-------------------

HSA ERBB2 DNA 10-15 (373) GTGCTGGGGATGGAGGAAGATGAGAATAGCCTTTGCTGACCGGGAAGGGGTCCGTGGTAAGGTGC

Cat ERBB2 DNA 10-15 (432) --ACACTCTTCCCACAGCGGTGCTTACTCGCTGACCCTTCAAGGGCTGGGCATCAGCTGGCTGGG

Cat ERBB2 DNA 10-15 Variant (432) --ACACTCTTCCCACAGCGGTGCTTACTCGCTGACCCTTCAAGGGCTGGGCATCAGCTGGCTGGG

HSA ERBB2 DNA 10-15 (438) CCACCTTTCTCCCATAGTGGCGCCTACTCGCTGACCCTGCAAGGGCTGGGCATCAGCTGGCTGGG

Cat ERBB2 DNA 10-15 (495) GCTGCGCTCGCTGCGGGAGCTGGGCAGTGGGCTGGCCCTCATCCACCGCAACTCCCGCCTCTGCT

Cat ERBB2 DNA 10-15 Variant (495) GCTGCGCTCGCTGCGGGAGCTGGGCAGTGGGCTGGCCCTCATCCACCGCAACTCCCGCCTCTGCT

HSA ERBB2 DNA 10-15 (503) GCTGCGCTCACTGAGGGAACTGGGCAGTGGACTGGCCCTCATCCACCATAACACCCACCTCTGCT

Cat ERBB2 DNA 10-15 (560) TCGTACACACGGTGCCCTGGGACCAGCTCTTCCGGAACCCCCACCAGGCCCTGCTCCACAGCGCC

Cat ERBB2 DNA 10-15 Variant (560) TCGTACACACGGTGCCCTGGGACCAGCTCTTCCGGAACCCCCACCAGGCCCTGCTCCACAGCGCC

HSA ERBB2 DNA 10-15 (568) TCGTGCACACGGTGCCCTGGGACCAGCTCTTTCGGAACCCGCACCAAGCTCTGCTCCACACTGCC

Cat ERBB2 DNA 10-15 (625) AACCGGCCAGAGGACGAGTGCGGTAAGACAGGGAGCCCAGCGCCACACGCTCCCCGTCTGCCGGC

Cat ERBB2 DNA 10-15 Variant (625) AACCGGCCAGAGGACGAGTGCGGTAAGACAGGGAGCCCAGCGCCACACGCTCCCCGTCTGCCGGC

HSA ERBB2 DNA 10-15 (633) AACCGGCCAGAGGACGAGTGTGGTAAGACAGGGAGCCCAGTGTG-CGCACTCCCCATCTGCCAGC

Cat ERBB2 DNA 10-15 (690) ACACAGGAGTGCCTGTGGGCCCCTAGCAGCAGCAATCTCGGACTTGTGCAGACTGCCCATCCCTG

Cat ERBB2 DNA 10-15 Variant (690) ACACAGGAGTGCCTGTGGGCCCCTAGCAGCAGCAATCTCGGACTTGTGCAGACTGCCCATCCCTG

HSA ERBB2 DNA 10-15 (697) ACACAGCAGTGCCCAGGGGGCCCTGGCAGCAGCGTTCTTGGACTTGTGCAGACTGCCCGTCTCTG

Cat ERBB2 DNA 10-15 (755) GTGCACCATTCTTGAC-----ACGGCTGTGGCTGGCTTGACTTCCTGGCATGGCTTCCGGCTGGG

Cat ERBB2 DNA 10-15 Variant (755) GTGCACCATTCTTGAC-----ACGGCTGTGGCTGGCTTGACTTCCTGGCATGGCTTCCGGCTGGG

HSA ERBB2 DNA 10-15 (762) -TGCACCCTTCTTGACTCAGCACAGCTCTGGCTGGCTTGGCCTCTTGGCATGGCTTCT--CTAGC

Cat ERBB2 DNA 10-15 (815) CCTGGCCACACTGCCTTGGCATCTGTGCCTTCCTTTTCCTCTCTGTCCCTGGAACCTCAG--CTC

Cat ERBB2 DNA 10-15 Variant (815) CCTGGCCACACTGCCTTGGCATCTGTGCCTTCCTTTTCCTCTCTGTCCCTGGAACCTCAG--CTC

HSA ERBB2 DNA 10-15 (824) TGGGTCCTACCTGCCTTGGCATC-----CTTCCCTC-CCCCTCTGTTTCTGAAATCTCAGAACTC

Cat ERBB2 DNA 10-15 (878) TTTCTTTCCCCGCATTGCCCGGCACCTATTCCCACCCCGTCCA**G**CCCACAGCC-TCCTC------

Cat ERBB2 DNA 10-15 Variant (878) TTTCTTTCCCCGCATTGCCCGGCACCTATTCCCACCCCGTCCA**C**CCCACAGCC-TCCTC------

HSA ERBB2 DNA 10-15 (883) TTCCTCTCCCTACATCGGCCC-CACCTGTCCCCACCCC-TCCAGCCCACAGCCATGCCCACAGCC

Cat ERBB2 DNA 10-15 (936) ----CCCTGGTTCATGTGGACCTGGAACCTTCCTTGAGTGTCCCCTCTAACCCCCTTCCTCTTCA

Cat ERBB2 DNA 10-15 Variant (936) ----CCCTGGTTCATGTGGACCTGGAACCTTCCTTGAGTGTCCCCTCTAACCCCCTTCCTCTTCA

HSA ERBB2 DNA 10-15 (946) AGTTCCCTGGTTCACTTGGACCTGGGGCCTCCCCTAAAAGTCCCCTGCGGTCCC-TTCCTCCTCA

Cat ERBB2 DNA 10-15 (997) CTGCAGCGGGTGAGGGCCTGGCCTGCTA**T**CCGCTGTGTGCCCACGGGCACTGCTGGGGTCCGGGA

Cat ERBB2 DNA 10-15 Variant (997) CTGCAGCGGGTGAGGGCCTGGCCTGCTA**C**CCGCTGTGTGCCCACGGGCACTGCTGGGGTCCGGGA

HSA ERBB2 DNA 10-15 (1010) CTGCAGTGGGCGAGGGCCTGGCCTGCCACCAGCTGTGCGCCCGAGGGCACTGCTGGGGTCCAGGG

Cat ERBB2 DNA 10-15 (1062) CCCACCCAGTGTGTCAACTGCAGCCAGTTCCTTCGGGGCCAGGAGTGCGTGGAGGAATGCCGAGT

Cat ERBB2 DNA 10-15 Variant (1062) CCCACCCAGTGTGTCAACTGCAGCCAGTTCCTTCGGGGCCAGGAGTGCGTGGAGGAATGCCGAGT

HSA ERBB2 DNA 10-15 (1075) CCCACCCAGTGTGTCAACTGCAGCCAGTTCCTTCGGGGCCAGGAGTGCGTGGAGGAATGCCGAGT

Cat ERBB2 DNA 10-15 (1127) A**T**TGCAGGGGTACGCGGGGCAGGGCAGGAGGGGCGGCCGGAGTGGGGCACGGGGGCTCCTTCCAG

Cat ERBB2 DNA 10-15 Variant (1127) A**C**TGCAGGGGTACGCGGGGCAGGGCAGGAGGGGCGGCCGGAGTGGGGCACGGGGGCTCCTTCCAG

HSA ERBB2 DNA 10-15 (1140) ACTGCAGGGGTATGAGGGGCGGAGGAG--AGGGTGGCTGGAGGGGTGCATGGGG-CTCCTCTCAG

Cat ERBB2 DNA 10-15 (1192) ACCCCCTCCTCAGCCATCCCTTCTGTCAGGCTTCCCCGGGAGTATGTGAAGGATAGGTTCTGTCT

Cat ERBB2 DNA 10-15 Variant (1192) ACCCCCTCCTCAGCCATCCCTTCTGTCAGGCTTCCCCGGGAGTATGTGAAGGATAGGTTCTGTCT

HSA ERBB2 DNA 10-15 (1202) ACCCCCTCACCA-CTGTCCCTTCTCTCAGGCTCCCCAGGGAGTATGTGAATGCCAGGCACTGTTT

Cat ERBB2 DNA 10-15 (1257) GCCATGCCACCCGGAGTGTCAGCCCCAGAATGGCTCAGTGACCTGCTTGGGCTCGGTGAGTTGCT

Cat ERBB2 DNA 10-15 Variant (1257) GCCATGCCACCCGGAGTGTCAGCCCCAGAATGGCTCAGTGACCTGCTTGGGCTCGGTGAGTTGCT

HSA ERBB2 DNA 10-15 (1266) GCCGTGCCACCCTGAGTGTCAGCCCCAGAATGGCTCAGTGACCTGTTTTGGACCGGTGAGCTGCT

Cat ERBB2 DNA 10-15 (1322) GGTGGGCTT-GATCGAGGTGGGTGGAGGGAAG**T**GAGGG-------CAGGGGCGGGGTGGGGTGGC

Cat ERBB2 DNA 10-15 Variant (1322) GGTGGGCTT-GATCGAGGTGGGTGGAGGGAAG**C**GAGGG-------CAGGGGCGGGGTGGGGTGGC

HSA ERBB2 DNA 10-15 (1331) GGCGGGCTCAGAGCTGGGTGGA-GGGGGGCAGCGAGGGGGATTGCCAGGGACTTGGCAGGATGGC

Cat ERBB2 DNA 10-15 (1379) CGG--GCACCTGGCCATG---TCCAGTAGAATGTCCCAGAAGAGGGCTCGGAGCTCAGCCCTGGT

Cat ERBB2 DNA 10-15 Variant (1379) CGG--GCACCTGGCCATG---TCCAGTAGAATGTCCCAGAAGAGGGCTCGGAGCTCAGCCCTGGT

HSA ERBB2 DNA 10-15 (1395) GAGATGCAGTAGGGTGTGCTATCTGGTAAAATATCCCTGGAGAGGGCTCAGCGCTCAGACCTGAA

Cat ERBB2 DNA 10-15 (1439) TGGCAGGGGGGTGTTAGGAGGCTGGCATGCTGGGGACTGCAAGTTGTGTAGTGGACCCTGTGACC

Cat ERBB2 DNA 10-15 Variant (1439) TGGCAGGGGGGTGTTAGGAGGCTGGCATGCTGGGGACTACAAGTTGTGTAGTGGACCCTGTGACC

HSA ERBB2 DNA 10-15 (1460) CAGCAACAGAGTGGCAGAAAAGGGGC---CTGGGG------------------GACACTGGGGCC

Cat ERBB2 DNA 10-15 (1504) CTTCAGACTATGCAAAGGTCCCGGGGAGGCCTGTGTCCTTGGCTGTG------GCTGTGCCAAGG

Cat ERBB2 DNA 10-15 Variant (1504) CTTCAGACTATGCAAAGGTCCCGGGGAGGCCTGTGTCCTTGGCTGTG------GCTGTGCCAAGG

HSA ERBB2 DNA 10-15 (1504) CTTCAGACTATGAAAAGGTTCTAAGGAGGTCTGTGTTGGTGGCTGTGACTGTGGCTGTGCTAGGG

Cat ERBB2 DNA 10-15 (1563) TGGTTAGACCTATGGGCTTAAGCATCAGACTCTCTGGATTCGGATCCAGGCCCTGCTTCCATCCT

Cat ERBB2 DNA 10-15 Variant (1563) TGGTTAGACCTATGGGCTTAAGCATCAGACTCTCTGGATTCGGATCCAGGCCCTGCTTCCATCCT

HSA ERBB2 DNA 10-15 (1569) TGGTGAGCCCTGTGGGCTCAGGCGTCAGACTACCTGGATTCAGACCCAGCTCCTGCTTCCAACCT

Cat ERBB2 DNA 10-15 (1628) TGGTTCGCTCATTTATAAAGTGAATATTGTA----------CATTG---GAATGTGGGAAGAATG

Cat ERBB2 DNA 10-15 Variant (1628) TGGTTCGCTCATTTATAAAGTGAATATTGTA----------CATTG---GAATGTGGGAAGAATG

HSA ERBB2 DNA 10-15 (1634) TGGTTTT-TTATTCCTAAAATGGGTATTGTAATAATACCTACCTTGCTGGGGTGTGGCAAGAATG

Cat ERBB2 DNA 10-15 (1680) AACTTAAAATAGGGTTTGGCACCGTGCC-CAAGCGATTTGCTGTGT---GTTAGTTAACTATTGT

Cat ERBB2 DNA 10-15 Variant (1680) AACTTAAAATAGGGTTTGGCACCGTGCC-CAAGCGATTTGCTGTGT---GTTAGTTAACTATTGT

HSA ERBB2 DNA 10-15 (1698) AAATTAAAC-AGGGCTTGGCACAGTGAAGCACGGGAAAGGCTTTCTACAGAGCAGTGACTGTTGT

Cat ERBB2 DNA 10-15 (1741) TACTTGCTGTTAT**A**TCTTAAGTAATACACTCCCCTG-CTAGA--CTTCCAGTTCTTCATCTGAAG

Cat ERBB2 DNA 10-15 Variant (1741) TACTTGCTGTTAT**C**TCTTAAGTAATACACTCCCCTG-CTAGA--CTTCCAGTTCTTCATCTGAAG

HSA ERBB2 DNA 10-15 (1762) TACTCGCTGTTACACCTTAGGTAATGCGTTTTCCTCTCTGGGTGCCTCCCATTTTCTGGCTCAAG

Cat ERBB2 DNA 10-15 (1803) CCTCCTGCCTGAGGATTGAGCTTTGAGCAGGGGAGGGGGGAGCTGGGTGAAGAGCAGGGGTGGCA

Cat ERBB2 DNA 10-15 Variant (1803) CCTCCTGCCTGAGGATTGAGCTTTGAGCAGGGGAGGGGGGAGCTGGGTGAAGAGCAGGGGTGGCA

HSA ERBB2 DNA 10-15 (1827) TC-CCTGCCC-AGGATCAAGCTTGGAGGAGGGCCCCGAGGGAGGGGCCACAGAGACTGGGTGAAG

Cat ERBB2 DNA 10-15 (1868) GACACGGCTGGT**C**TGGGCTGTTCCAGGAGCAAGGCGAAAGT-GCTGT**G**GAGTGGCCATGGGAAGC

Cat ERBB2 DNA 10-15 Variant (1868) GACACGGCTGGT**T**TGGGCTGTTCCAGGAGCAAGGCGAAAGT-GCTGT**C**GAGTGGCCATGG**C**AAGC

HSA ERBB2 DNA 10-15 (1890) AGCAAGGGTGTT------TGTCCCAGGAGCATGGCGAAAATTGCTGCTGGGTGGCCTTGGGAAGC

Cat ERBB2 DNA 10-15 (1932) ACGAAGGGGACACAA----GGGCATG-TCCTGCTGCCCTGGAGGTGTTGATGCTAGCCCCCTTCC

Cat ERBB2 DNA 10-15 Variant (1932) ACGAAGGGGACACAA----GGGCATG-TCCTGCTGCCCTGGAGGTGTTGATGCTAGCCCCCTTCC

HSA ERBB2 DNA 10-15 (1949) ACAAAGGGGACCCAACTAAGGGCCTGATCCTACTGCCCTGGGGGTGTCAGTGCCAGCCCCCCACA

Cat ERBB2 DNA 10-15 (1992) AA**A**TCCTTCTTCCCTTCCCAGGAAGCTGACCAGTGTGTGGCCTGT**G**CCC**A**CTACAAGGACCCTCC

Cat ERBB2 DNA 10-15 Variant (1992) AA**-**TCCTTCTTCCCTTCCCAGGAAGCTGACCAGTGTGTGGCCTGT**C**CCC**C**CTACAAGGACCCTCC

HSA ERBB2 DNA 10-15 (2014) AATCTTTTCTGCCCCCCCCAGGAGGCTGACCAGTGTGTGGCCTGTGCCCACTATAAGGACCCTCC

Cat ERBB2 DNA 10-15 (2057) TTTCTGTG**T**GGCTCGCTGCCCCAGTGGGGTGAAACCTGACCTCTCCTTCATGCCCATCTGGAAGT

Cat ERBB2 DNA 10-15 Variant (2057) TTTCTGTG**C**GGCTCGCTGCCCCAGTGGGGTGAAACCTGACCTCTCCTTCATGCCCATCTGGAAGT

HSA ERBB2 DNA 10-15 (2079) CTTCTGCGTGGCCCGCTGCCCCAGCGGTGTGAAACCTGACCTCTCCTACATGCCCATCTGGAAGT

Cat ERBB2 DNA 10-15 (2122) TCGCAGATGAGGAGGGCACGTGCCAGCCATGCCCCATCAACTGCACCCACTC----

Cat ERBB2 DNA 10-15 Variant (2122) TCGCAGATGAGGAGGGCACGTGCCAGCCATGCCCCATCAACTGCACCCACTC----

HSA ERBB2 DNA 10-15 (2144) TTCCAGATGAGGAGGGCGCATGCCAGCCTTGCCCCATCAACTGCACCCACTCTCCA

**Legend:** Multi-alignment between reference (Cat *ERBB2* DNA 10-15) and variant (Cat *ERBB2* DNA 10-15 variant) cat *ERBB2* DNA sequences and the corresponding Human *ERBB2* variant 2 mRNA sequence (Human *ERBB2* mRNA 10-15). The genomic SVs are black highlight. (N) Multi-allelic SV present in genomic position 271 (g.271 T>G and g.271 T>A).
