# Supplementary material for: ERBB2 in Cat Mammary Neoplasias Disclosed a Positive Correlation between RNA and Protein Low Expression Levels: A Model for erbB-2 Negative Human Breast Cancer
Source: PLoS One. 2013 Dec 26;8(12):e83673. doi: 10.1371/journal.pone.0083673 (PMC3873372; doi:10.1371/journal.pone.0083673)
Supplement: Figure S2 — Align study of ERBB2 partial coding sequence corresponding to exons 10–15 transcripts. Multi-alignment between wild-type sequence (Cat ERBB2 CDS 10–15 wt) and variant (Cat ERBB2 CDS 10–15 variant) cat ERBB2 cDNA sequence and the corresponding Human ERBB2 variant 2 mRNA sequence (Human ERBB2 mRNA 10–15). The cat ERBB2 CDS sequences were obtained by introns sequences deletion from the reference and variant sequences (submitted to GeneBank). The nsSVs detected in CDS are in red. The synonymous SVs detected in CDS are in green. Adobe (.PDF); paper size A4. (DOC) [file pone.0083673.s002.doc]

**Additional Figure, Santos *et al*.; Adobe (.PDF); paper size A4**

**Figure S2: Align study of *ERBB2* partial coding sequence corresponding to exons 10-15 transcripts.**

Cat *ERBB2* CDS wt 10-15 (1) GGACCCAGCCTCCAACACTGCCCCCCTGCAGCCTGAGCAGCTCAGAGTGT

Cat *ERBB2* CDS 10-15 variant (1) GGACCCAGCCTCCAACACTGCCCCCCTGCAGCCTGAGCAGCTCAGAGTGT

Human *ERBB2* mRNA 10-15 (1) GGACCCAGCCTCCAACACTGCCCCGCTCCAGCCAGAGCAGCTCCAAGTGT

Cat *ERBB2* CDS wt 10-15 (51) TTGAGGCTCTGGAGGAGATTACAGGTTACCTGTACATCTCAGCGTGGCCA

Cat *ERBB2* CDS 10-15 variant (51) TTGAGGCTCTGGAGGAGATTACAGGTTACCTGTACATCTCAGCGTGGCCA

Human *ERBB2* mRNA 10-15 (51) TTGAGACTCTGGAAGAGATCACAGGTTACCTATACATCTCAGCATGGCCG

Cat *ERBB2* CDS wt 10-15 (101) GACAGCTTGCCTAACCTCAGTGTCTTCCAGAACCTCA**G**AG**T**GATCCGGGG

Cat *ERBB2* CDS 10-15 variant (101) GACAGCTTGCCTAACCTCAGTGTCTTCCAGAACCTCA**A**AG**A**GATCCGGGG

Human *ERBB2* mRNA 10-15 (101) GACAGCCTGCCTGACCTCAGCGTCTTCCAGAACCTGCAAGTAATCCGGGG

Cat *ERBB2* CDS wt 10-15 (151) CCGAGTTCTGCATGACGGTGCTTACTCGCTGACCCTTCAAGGGCTGGGCA

Cat *ERBB2* CDS 10-15 variant (151) CCGAGTTCTGCATGACGGTGCTTACTCGCTGACCCTTCAAGGGCTGGGCA

Human *ERBB2* mRNA 10-15 (151) ACGAATTCTGCACAATGGCGCCTACTCGCTGACCCTGCAAGGGCTGGGCA

Cat *ERBB2* CDS wt 10-15 (201) TCAGCTGGCTGGGGCTGCGCTCGCTGCGGGAGCTGGGCAGTGGGCTGGCC

Cat *ERBB2* CDS 10-15 variant (201) TCAGCTGGCTGGGGCTGCGCTCGCTGCGGGAGCTGGGCAGTGGGCTGGCC

Human *ERBB2* mRNA 10-15 (201) TCAGCTGGCTGGGGCTGCGCTCACTGAGGGAACTGGGCAGTGGACTGGCC

Cat *ERBB2* CDS wt 10-15 (251) CTCATCCACCGCAACTCCCGCCTCTGCTTCGTACACACGGTGCCCTGGGA

Cat *ERBB2* CDS 10-15 variant (251) CTCATCCACCGCAACTCCCGCCTCTGCTTCGTACACACGGTGCCCTGGGA

Human *ERBB2* mRNA 10-15 (251) CTCATCCACCATAACACCCACCTCTGCTTCGTGCACACGGTGCCCTGGGA

Cat *ERBB2* CDS wt 10-15 (301) CCAGCTCTTCCGGAACCCCCACCAGGCCCTGCTCCACAGCGCCAACCGGC

Cat *ERBB2* CDS 10-15 variant (301) CCAGCTCTTCCGGAACCCCCACCAGGCCCTGCTCCACAGCGCCAACCGGC

Human *ERBB2* mRNA 10-15 (301) CCAGCTCTTTCGGAACCCGCACCAAGCTCTGCTCCACACTGCCAACCGGC

Cat *ERBB2* CDS wt 10-15 (351) CAGAGGACGAGTGCGCGGGTGAGGGCCTGGCCTGCTA**T**CCGCTGTGTGCC

Cat *ERBB2* CDS 10-15 variant (351) CAGAGGACGAGTGCGCGGGTGAGGGCCTGGCCTGCTA**C**CCGCTGTGTGCC

Human *ERBB2* mRNA 10-15 (351) CAGAGGACGAGTGTGTGGGCGAGGGCCTGGCCTGCCACCAGCTGTGCGCC

Cat *ERBB2* CDS wt 10-15 (401) CACGGGCACTGCTGGGGTCCGGGACCCACCCAGTGTGTCAACTGCAGCCA

Cat *ERBB2* CDS 10-15 variant (401) CACGGGCACTGCTGGGGTCCGGGACCCACCCAGTGTGTCAACTGCAGCCA

Human *ERBB2* mRNA 10-15 (401) CGAGGGCACTGCTGGGGTCCAGGGCCCACCCAGTGTGTCAACTGCAGCCA

Cat *ERBB2* CDS wt 10-15 (451) GTTCCTTCGGGGCCAGGAGTGCGTGGAGGAATGCCGAGTA**T**TGCAGGGGC

Cat *ERBB2* CDS 10-15 variant (451) GTTCCTTCGGGGCCAGGAGTGCGTGGAGGAATGCCGAGTA**C**TGCAGGGGC

Human *ERBB2* mRNA 10-15 (451) GTTCCTTCGGGGCCAGGAGTGCGTGGAGGAATGCCGAGTACTGCAGGGGC

Cat *ERBB2* CDS wt 10-15 (501) TTCCCCGGGAGTATGTGAAGGATAGGTTCTGTCTGCCATGCCACCCGGAG

Cat *ERBB2* CDS 10-15 variant (501) TTCCCCGGGAGTATGTGAAGGATAGGTTCTGTCTGCCATGCCACCCGGAG

Human *ERBB2* mRNA 10-15 (501) TCCCCAGGGAGTATGTGAATGCCAGGCACTGTTTGCCGTGCCACCCTGAG

Cat *ERBB2* CDS wt 10-15 (551) TGTCAGCCCCAGAATGGCTCAGTGACCTGCTTGGGCTCGGAAGCTGACCA

Cat *ERBB2* CDS 10-15 variant (551) TGTCAGCCCCAGAATGGCTCAGTGACCTGCTTGGGCTCGGAAGCTGACCA

Human *ERBB2* mRNA 10-15 (551) TGTCAGCCCCAGAATGGCTCAGTGACCTGTTTTGGACCGGAGGCTGACCA

Cat *ERBB2* CDS wt 10-15 (601) GTGTGTGGCCTGT**G**CCC**A**CTACAAGGACCCTCCTTTCTGTG**T**GGCTCGCT

Cat *ERBB2* CDS 10-15 variant (601) GTGTGTGGCCTGT**C**CCC**C**CTACAAGGACCCTCCTTTCTGTG**C**GGCTCGCT

Human *ERBB2* mRNA 10-15 (601) GTGTGTGGCCTGTGCCCACTATAAGGACCCTCCCTTCTGCGTGGCCCGCT

Cat *ERBB2* CDS wt 10-15 (651) GCCCCAGTGGGGTGAAACCTGACCTCTCCTTCATGCCCATCTGGAAGTTC

Cat *ERBB2* CDS 10-15 variant (651) GCCCCAGTGGGGTGAAACCTGACCTCTCCTTCATGCCCATCTGGAAGTTC

Human *ERBB2* mRNA 10-15 (651) GCCCCAGCGGTGTGAAACCTGACCTCTCCTACATGCCCATCTGGAAGTTT

Cat *ERBB2* CDS wt 10-15 (701) GCAGATGAGGAGGGCACGTGCCAGCCATGCCCCATCAACTGCACCCACTC

Cat *ERBB2* CDS 10-15 variant (701) GCAGATGAGGAGGGCACGTGCCAGCCATGCCCCATCAACTGCACCCACTC

Human *ERBB2* mRNA 10-15 (701) CCAGATGAGGAGGGCGCATGCCAGCCTTGCCCCATCAACTGCACCCACTC

Legend: Multi-alignment between wild type sequence (Cat *ERBB2* CDS 10-15 wt) and variant (Cat *ERBB2* CDS 10-15 variant) cat *ERBB2* cDNA sequence and the corresponding Human *ERBB2* variant 2 mRNA sequence (Human *ERBB2* mRNA 10-15). The cat *ERBB2* CDS sequences were obtained by introns sequences deletion from the reference and variant sequences (submitted to GeneBank). The nsSVs detected in CDS are in red. The synonymous SVs detected in CDS are in orange and underline.
