# Supplementary material for: ERBB2 in Cat Mammary Neoplasias Disclosed a Positive Correlation between RNA and Protein Low Expression Levels: A Model for erbB-2 Negative Human Breast Cancer
Source: PLoS One. 2013 Dec 26;8(12):e83673. doi: 10.1371/journal.pone.0083673 (PMC3873372; doi:10.1371/journal.pone.0083673)
Supplement: Figure S3 — Align study of ErbB-2 partial protein sequence corresponding to exons 10–15 translation. Multi-alignment between wild-type (Cat erbB-2 10–15 protein wt) and variant cat erbB-2 protein sequence and with the corresponding Human erbB-2 partial protein sequence. The Human sequence correspond to part of the chain A from the 3BE1 model. The amino-acid changes detected are indicated in red. Adobe (.PDF); paper size 21×15 cm. (DOC) [file pone.0083673.s003.doc]

**Additional Figure Santos *et al*.; Word (.doc); Paper size A4**

**Figure S3: Align study of ErbB-2 partial protein sequence corresponding to exons 10-15 translation.**

Cat erbB-2 10-15 prot wt (1) DPASNTAPLQPEQLRVFEALEEITGYLYISAWPDSLPNLSVFQNL**RV**IRG

Cat erbB-2 10-15 prot variant (1) DPASNTAPLQPEQLRVFEALEEITGYLYISAWPDSLPNLSVFQNL**KE**IRG

Human erbB-2(3BE/1A)10-15 prot (1) SFDGNTAPLQPEQLQVFETLEEITGYLYISAWPDSLPDLSVFQNLQVIRG

Cat erbB-2 10-15 prot wt (51) RVLHDGAYSLTLQGLGISWLGLRSLRELGSGLALIHRNSRLCFVHTVPWD

Cat erbB-2 10-15 prot variant (51) RVLHDGAYSLTLQGLGISWLGLRSLRELGSGLALIHRNSRLCFVHTVPWD

Human erbB-2(3BE1/A)10-15 prot (51) RILHNGAYSLTLQGLGISWLGLRSLRELGSGLALIHHNTHLCFVHTVPWD

Cat erbB-2 10-15 prot wt (101) QLFRNPHQALLHSANRPEDECAGEGLACYPLCAHGHCWGPGPTQCVNCSQ

Cat erbB-2 10-15 prot variant (101) QLFRNPHQALLHSANRPEDECAGEGLACYPLCAHGHCWGPGPTQCVNCSQ

Human erbB-2(3BE1/A)10-15 prot (101) QLFRNPHQALLHTANRPEDECVGEGLACHQLCARGHCWGPGPTQCVNCSQ

Cat erbB-2 10-15 prot wt (151) FLRGQECVEECRVLQGLPREYVKDRFCLPCHPECQPQNGSVTCLGSEADQ

Cat erbB-2 10-15 prot variant (151) FLRGQECVEECRVLQGLPREYVKDRFCLPCHPECQPQNGSVTCLGSEADQ

Human erbB-2(3BE1/A)10-15 prot (151) FLRGQECVEECRVLQGLPREYVNARHCLPCHPECQPQNGSVTCFGPEADQ

Cat erbB-2 10-15 prot wt (201) CVAC**AH**YKDPPFC**V**ARCPSGVKPDLSFMPIWKFADEEGTCQPCPINCTH

Cat erbB-2 10-15 prot variant (201) CVAC**PP**YKDPPFC**A**ARCPSGVKPDLSFMPIWKFADEEGTCQPCPINCTH

Human erbB-2(3BE1/A)10-15 prot (201) CVACAHYKDPPFCVARCPS----------IWKFPDEEGACQPCPIN---

**Legend**: Multi-alignment between wild type (Cat erbB-2 10-15 protein wt) and variant cat erbB-2 protein sequence and with the corresponding Human erbB-2 partial protein sequence. The Human sequence correspond to part of the chain A from the 3BE1 model. The amino-acid changes detected are indicated in red.
