# Supplementary material for: ERBB2 in Cat Mammary Neoplasias Disclosed a Positive Correlation between RNA and Protein Low Expression Levels: A Model for erbB-2 Negative Human Breast Cancer
Source: PLoS One. 2013 Dec 26;8(12):e83673. doi: 10.1371/journal.pone.0083673 (PMC3873372; doi:10.1371/journal.pone.0083673)
Supplement: Figure S4 — Western blot with the CBE356 and CB11 anti-erbB2 antibodies. Reactivity observed by western immunoblot with the CBE356 and CB11 anti-erbB2 antibodies, in 3 feline mammary tissues. Lane 1: non-neoplastic cat mammary tissues; Lanes 2-3: cat mammary lesion samples. The expected molecular weight (185 kDa) of the reactive protein band, was determined by comparison, with the molecular weights of the standard Protein Marker (PM). Adobe (.PDF); paper size 21×15 cm. (PDF) [file pone.0083673.s004.pdf]

Additional Figure S4, Santos *et al*.

Figure S4: Reactivity observed by western immunoblot with the CBE356 and CB11 anti-erbB2 antibodies, in 3 feline mammary tissues.

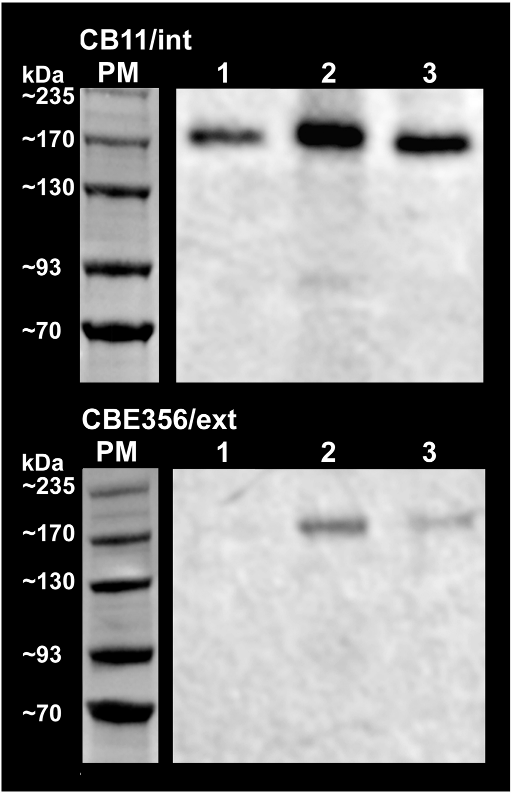

Legend: The expected molecular weight (185 kDa) of the reactive protein band, was determined by comparison, with the molecular weights of the standard Protein Marker (PM). Lane 1: non-neoplastic cat mammary tissues; Lanes 2-3: cat mammary lesion samples. .
