# Supplementary material for: ERBB2 in Cat Mammary Neoplasias Disclosed a Positive Correlation between RNA and Protein Low Expression Levels: A Model for erbB-2 Negative Human Breast Cancer
Source: PLoS One. 2013 Dec 26;8(12):e83673. doi: 10.1371/journal.pone.0083673 (PMC3873372; doi:10.1371/journal.pone.0083673)
Supplement: Figure S5 — ERBB2 DNA copy number quantification: standard curve construct and copy number/haploid genome assessment. (A) Standard curve constructed with a 2-fold serial dilution series of the plasmid DNA with the ERBB2 insert, ranging from 3.12×10^7^ to 1.95×10^6^ copies (5 points series dilutions, each point in triplicate). The Efficiency of the PCR reaction was 98% (R2 = 0.996). (B) 10 normal and 10 mammary lesions were subjected to real time quantitative PCR for gene copy number evaluation. In all cases we didn't detect any gene amplification. (IHC) Immunohistochemistry. Adobe (.PDF); paper size 21×15 cm. (PDF) [file pone.0083673.s005.pdf]

Additional Figure Santos *et al.*; PDF; Paper size A4

Figure S5: *ERBB2* DNA copy number quantification: standard curve construct and copy number / haploid genome assessment

A

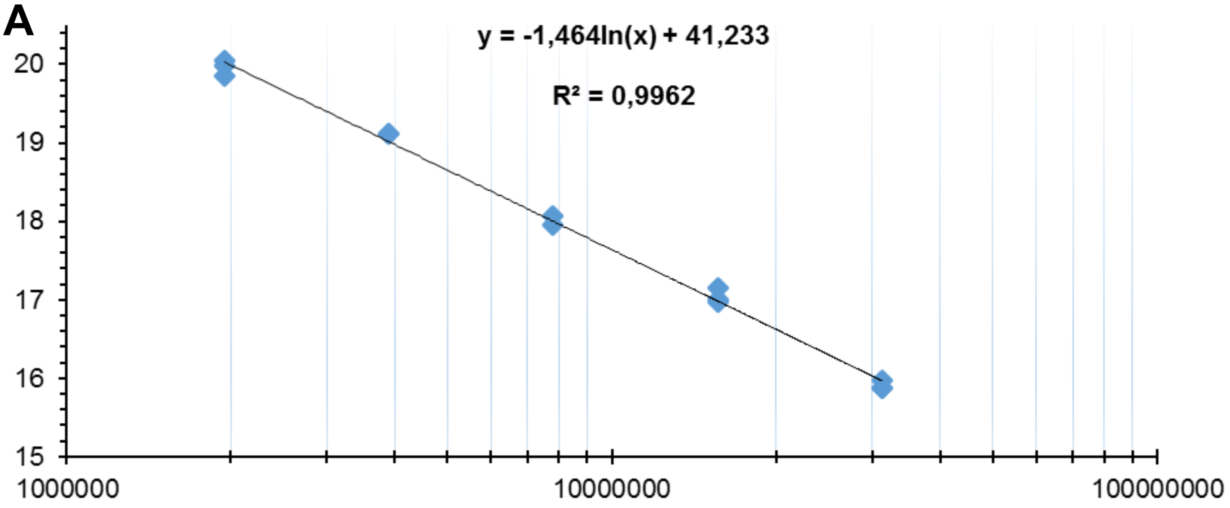

B

Normal Sample	*ERBB2* copy number/haploid genome		Lesion Sample ID	IHC score CB11	IHC score CBE356	*ERBB2* copy number/haploid genome	
**1**	0,943	1	**B_L1**	+++	+++	1,061	1
**2**	1,188	1	**B-L2**	+++	+++	1,414	1
**3**	1,134	1	**B_L5**	++	+++	0,685	1
**4**	1,019	1	**ML1**	0	0	1,092	1
**5**	1,030	1	**ML3**	0	++	1,179	1
**6**	0,676	1	**ML6**	0	+	1,251	1
**7**	1,244	1	**ML15**	0	+	0,945	1
**8**	1,140	1	**ML16**	0	+	1,008	1
**9**	1,205	1	**ML17**	0	+	1,624	2
**10**	0,718	1	**ML19**	+++	+++	0,547	1

Legend: (A) Standard curve constructed with a 2-fold serial dilution series of the plasmid DNA with the *ERBB2* insert, ranging from $3{,}12\times10^{7}$ to $1{,}95\times10^{6}$ copies (5 points series dilutions, each point in triplicate). The Efficiency of the PCR reaction was 98% (R^2=0,996). **(B)** 10 normal and 10 mammary lesions were subjected to real time quantitative PCR for gene copy number evaluation. In all cases we didn't detect any gene amplification. (IHC) Immunohistochemistry.
