# Supplementary material for: ERBB2 in Cat Mammary Neoplasias Disclosed a Positive Correlation between RNA and Protein Low Expression Levels: A Model for erbB-2 Negative Human Breast Cancer
Source: PLoS One. 2013 Dec 26;8(12):e83673. doi: 10.1371/journal.pone.0083673 (PMC3873372; doi:10.1371/journal.pone.0083673)
Supplement: Table S1 — Summary of the clinicopathological evaluation and clinical outcome of the cat mammary benign, malign and metastatic lesions study in the present work. Tumour identification (ID) in 3 categories: (BL) benign lesions; (MaL) primary malign lesion; (MeL) metastasis lesion. (neo) neoplastic; (hip) hyperplasia; (LN) Lynph node; (rec) recurrence; (NE) No evidence; (NA/E) Not applicable/evaluated. Clinical outcome evaluation: (DOD) dead of disease; (DC) developed a carcinoma (NED): no evidence of the disease; (*NED) no evidence of the disease for more than 2 years; (DOC) dead of other causes. (#) Histological classification in accord with Misdorp et al. (1999) [83]. The evaluation grade is in accord with Gimenez et al. (2010) [85] and Misdorp W. (2002) [6] (describe in Table S2). Word (.doc); Page size 33×33 cm. (DOC) [file pone.0083673.s006.doc]

**Additional file; Table S1; Word (.doc); Page size 33 x 33 cm**

**Table S1: Summary of the clinicopathological evaluation and clinical outcome of the cat mammary benign, malign and metastatic lesions study in the present work.**

| **Tumour**  **ID** | **Histological**  **Classification (#)** | **Race** | **Ovary**  **hysterectomy** | **Hormonal treatment** | **Age**  **(years-old)** | **Lesion diameter size (cm)** | **Number**  **mitosis / field** | **Nuclear / Cellular polymorphism** | **Nº of lesions** | **Lynph node invasion** | **Vascular infiltration** | **Necrosis** | **Clinical Outcome** |
| --- | --- | --- | --- | --- | --- | --- | --- | --- | --- | --- | --- | --- | --- |
| BL1 (hip.) | Fibroadenomatous Hyperplasia | Domestic shorthaired | Yes | Yes | 6 | < 2 | 0-7 | No evidence | Multiple | NA/E | NA/E | No evidence | DC |
| BL2 (neo.) | Fibroadenoma | Domestic shorthaired |  |  | 10 | > 3 |  | No evidence | Multiple | NA/E | NA/E | NA/E | *NED |
| BL3 (hip.) | Hyperplasia | Domestic shorthaired | No |  | 13 | < 2 |  | No evidence | Multiple | NA/E | No evidence | No evidence |  |
| BL4 (neo.) | Fibroadenoma |  |  |  |  |  |  | No evidence | Multiple | NA/E | NA/E | NA/E |  |
| BL5 (hip.) | Fibroadenomatous Hyperplasia | Domestic shorthaired | No | Yes | 1 | 2 to 3 | 0-7 | No evidence | Multiple | NA/E | No evidence | No evidence | *NED |
| BL6 (hip.) | Lobular Hyperplasia (epiteliosis) | Domestic shorthaired | Yes | No | 12 | < 2 |  | No evidence | Multiple | NA/E | No evidence | No evidence | *NED |
| MaL1 | Tubular | Domestic shorthaired | Yes | No | 8 | 2 to 3 | 0-7 | Marked | Single | No evidence | No evidence | Evidence | *NED |
| MaL2 | Tubulopapillary | Siamese | Yes | No |  | < 2 |  |  | Single | NA/E | Evidence | Evidence | DOD |
| MaL3 | Tubulopapillary/solid | Domestic shorthaired | Yes | No | 10 | 2 to 3 |  | Marked | Single | Evidence | Evidence | Evidence | DOD |
| MaL4 | Carcinoma |  |  |  | 15 |  |  |  | Multiple | NA/E | NA/E | NA/E |  |
| MaL5 | Carcinoma |  |  |  |  |  |  |  | Multiple | NA/E | NA/E | NA/E |  |
| MaL6 | Tubulopapillary (hiper/epit) | Domestic shorthaired | No | Yes | 14 | < 2 | 0-7 | Low | Multiple | NA/E | No evidence | No evidence | DOC |
| MaL7 | Carcinoma |  |  |  |  |  |  |  |  | Evidence | NA/E | NA/E |  |
| MaL8 | Papillary |  |  |  | 15 | < 2 |  |  | Multiple | Evidence | Evidence | NA/E |  |
| MaL9 | Tubulopapillary | Domestic shorthaired | No | Yes | 6 | < 2 | 0-7 | Low | Multiple | No evidence | No evidence | No evidence |  |
| MaL10 | Tubulopapillary/solid | Domestic shorthaired |  |  | 15 | > 3 | 8 - 14 | Moderated | Multiple | No evidence | NA/E | Evidence | DOD |
| MaL11 | Tubular/solid | Indeterminate | No | Yes | 10 | > 3 | 0-7 |  | Multiple | Evidence | Evidence | Evidence | DOD |
| MaL12 | Papillary | Domestic shorthaired |  |  | 6 | > 3 |  |  | Multiple | NA/E | NA/E | NA/E |  |
| MaL13 | Papillary | Domestic shorthaired | Yes | Yes | 7 | < 2 | 0-7 | Moderated | Multiple | No evidence | NA/E | No evidence | *NED |
| MaL14 | Cribiform | Siamese |  | No | 12 | < 2 | 0-7 | Marked | Multiple | Evidence | NA/E | Evidence | DOD |
| MaL15 | Tubulopapillary (GII) | Domestic shorthaired | No |  |  | > 3 | >15 | Moderated | Multiple | Evidence | Evidence | Evidence | DOC |
| MaL16 | Tubulopapillary (GII) | Domestic shorthaired | No | Yes | 9 | 2 to 3 | 8 - 14 | Marked | Multiple | Evidence | Evidence | Evidence | DOD |
| MaL17 | Tubulopapillary (MaL16 rec) | Domestic shorthaired | Yes | No | 10 | < 2 | 0-7 | Marked | Multiple | NA/E | NA/E | Evidence | DOD |
| MaL18 | Tubulopapillary (GI/GII) | Domestic shorthaired |  |  | 9 | 2 to 3 | 8 - 14 | Marked | Multiple | NA/E | NA/E | Evidence | NED |
| MaL19 | Tubulopapillary (GI) / mucinous (GII) | Domestic Shorthaired x Siamese | No | Yes | 17 | 2 to 3 | 8 - 14 | Moderated | Multiple | Evidence | Evidence | Evidence | DOD |
| MaL20 | Tubulopapillary (GI/II) | Domestic shorthaired | No | Yes | 10 | > 3 | >15 | Moderated | Multiple | Evidence | Evidence | Evidence | DOD |
| MaL21 | Cribiform (MaL20 rec) | Domestic shorthaired | Yes | No | 10 | 2 to 3 | 8 - 14 | Marked | Multiple | Evidence | Evidence | Evidence | DOD |
| MaL22 | Tubulopapillary | Domestic shorthaired | No | Yes | 8 | < 2 | 0-7 | Moderated | Single | Evidence | Evidence | NA/E | NED |
| MeL1 | LN Metastasis |  |  |  |  |  |  |  |  |  |  |  |  |
| MeL2 | LN Metastasis |  |  |  |  |  |  |  |  |  |  |  | DOD |
| MeL3 | Pulmonary Metastasis |  |  |  |  |  |  |  |  |  |  |  | DOD |
| MeL4 | LN Metastasis (MaL22) |  |  |  | 8 |  |  |  |  |  |  |  | NED |
| MeL5 | Pulmonary Metastasis (MaL14 rec) |  |  |  | 12 |  |  |  |  |  |  |  | DOD |
| MeL6 | Pleura Metastasis (MaL14 rec) |  |  |  |  |  |  |  |  |  |  |  |  |
| MeL7 | Mesentery Metastasis (MaL14 rec) |  |  |  |  |  |  |  |  |  |  |  |  |
| MeL8 | Liver Metastasis (MaL14 rec) |  |  |  |  |  |  |  |  |  |  |  |  |
| MeL9 | Spleen Metastasis (MaL14 rec) |  |  |  |  |  |  |  |  |  |  |  |  |
| MeL10 | Tracheal Metastasis (MaL14 rec) |  |  |  |  |  |  |  |  |  |  |  |  |
| MeL11 | Liver Metastasis (MaL17) |  |  |  | 10 |  |  |  |  |  |  |  | DOD |
| MeL12 | Pulmonary Metastasis (MaL17) |  |  |  |  |  |  |  |  |  |  |  |  |
| MeL13 | LN Metastasis (MaL16) |  |  |  | 9 |  |  |  |  |  |  |  | DOD |
| MeL14 | LN Metastasis (MaL19) |  |  |  | 17 |  |  |  |  |  |  |  | DOD |
| MeL15 | LN Metastasis (MaL21) |  |  |  | 10 |  |  |  |  |  |  |  | DOD |

**Legend:** Tumour identification (ID) in 3 categories: (BL) benign lesions; (MaL) primary malign lesion; (MeL) metastasis lesion. (neo) neoplastic; (hip) hyperplasia; (LN) Lynph node; (rec) recurrence; (NE) No evidence; (NA/E) Not applicable/evaluated. Clinical outcome evaluation: (DOD) dead of disease; (DC) developed a carcinoma (NED): no evidence of the disease; (*NED) no evidence of the disease for more than 2 years; (DOC) dead of other causes; (#) Histological classification in accord with Misdorp *et al*. (1999) [83]. The evaluation grade is in accord with Gimenez *et al*. (2010) [85] and Misdorp W. (2002) [6] (describe in Table S2).
