# Supplementary material for: ERBB2 in Cat Mammary Neoplasias Disclosed a Positive Correlation between RNA and Protein Low Expression Levels: A Model for erbB-2 Negative Human Breast Cancer
Source: PLoS One. 2013 Dec 26;8(12):e83673. doi: 10.1371/journal.pone.0083673 (PMC3873372; doi:10.1371/journal.pone.0083673)
Supplement: Table S2 — Cat mammary lesions clinical and histological grading evaluation features and other prognostic factors. Accordingly with Gimenez et al. (2010) (*) [85] and Misdorp, W. (2002) (#) [6]. The scores grading presuppose that, for each clinicopathological characteristic, a higher value corresponded to a worse prognostic/clinical evaluation. Word (.doc); Page size A4. (DOC) [file pone.0083673.s007.doc]

**Additional data, Santos *et al*. Word (.doc); Page size A4**

**Table S2: Cat mammary lesions clinical and histological grading evaluation features and other prognostic factors.**

| **Histological Grading *** |  |  |
| --- | --- | --- |
| **Nuclear/cellular pleomorphism** * |  |  |
| Presence | Differential Grade: | Score |
| No evidence | Well | 1 |
| Low | Well | 2 |
| Moderated | Moderated | 3 |
| Marked | Poorly | 4 |
| **Mitotic Count *** |  |  |
| Number of mitoses per field area | Differential Grade | Score |
| 0 to 7 | Well | 1 |
| 8 to 14 | Moderated | 2 |
| >15 | Poorly | 3 |
| **Clinical Staging *** |  |  |
| **Primary Tumor Size *** |  |  |
| Diameter (cm) | Size Grade | Score |
| < 2 | T1 | 1 |
| 2 to 3 | T2 | 2 |
| > 3 | T3 | 3 |
| **Regional Lymph Node Metastasis *** |  |  |
| Presence | Presence grade | Score |
| No evidence | N0 | 1 |
| Evidence | N1 | 2 |
| **Other prognostic factors #** |  |  |
| **Vascular Infiltration #** |  |  |
| Presence | Presence grade | Score |
| No evidence | V0 | 1 |
| Evidence | V1 | 2 |
| **Necrosis #** |  |  |
| Presence | Presence grade | Score |
| No evidence | Ne0 | 1 |
| Evidence | Ne1 | 2 |
| **Age #** | Grade in years | Score |
|  | Ascending numerical order | |

**Legend:** Accordingly with Gimenez et al. (2010) (*) [85] and Misdorp, W. (2002) (#) [6]. The scores grading presuppose that, for each clinicopathological characteristic, a higher value corresponded to a worse prognostic/clinical evaluation.
