# Supplementary material for: ERBB2 in Cat Mammary Neoplasias Disclosed a Positive Correlation between RNA and Protein Low Expression Levels: A Model for erbB-2 Negative Human Breast Cancer
Source: PLoS One. 2013 Dec 26;8(12):e83673. doi: 10.1371/journal.pone.0083673 (PMC3873372; doi:10.1371/journal.pone.0083673)
Supplement: Table S3 — Analysis of sequence variants detected in the Cat ERBB2 gene fragment between exons 10 to 15. A total of 30 SVs were detected with dissimilar observation of 15 SVs in normal and CML samples. The minor allele and the genotype heterozygosis frequencies (%) were calculated individually for each SV. The Hardy-Weinberg equilibrium is indicated by the frequency value (p-value) calculated by the Chi-square test. The SVs that present p<0.05 are considered to present HWE deviation. The probable amino-acid changes are indicated whit the respective number of the amino-acid in the cat erbB-2_10/15 protein sequence. Two synonymous (Syn.) and five non-Synonymous (Non-Syn.) variations were detected in exonic positions. The zero percentage values were removed for better visualization. Footnotes: (*) 6 SVs only in normal samples; (#) 9 SVs only in mammary lesions samples; (a) SVs with significant values of allelic association with the CMLs; (g) SVs with significant values of genotypic association with the CMLs. Word (.doc); paper size: 29×25 cm. (DOC) [file pone.0083673.s008.doc]

**Additional data, Santos *et al*.**

**Table S3. Analysis of sequence variants detected in the Cat *ERBB2* gene fragment between exons 10 to 15.**

| **Cat ERBB2 gDNA** | | | | **Minor Allele Frequency (%)** | | | | | **Genotype Heterozygosis (%)** | | | | | **Allelic HWE (p value)** | | | **erbB-2 Protein Variation** | | |
| --- | --- | --- | --- | --- | --- | --- | --- | --- | --- | --- | --- | --- | --- | --- | --- | --- | --- | --- | --- |
| **Sequence Variants** | **notes** | | **Minor Allele** | **Normal** | **Benign** | **Malign** | **Metas.** | **CMLs’** | **Normal** | **Benign** | **Malign** | **Metas.** | **CMLs’** | **Normal** | **CMLs’** | **Total** | **Function** | **Type** | **AA**  **Change** |
| g.226 G>A | (*) | A | | 7.69 | - | - | - | - | 15.38 | - | - | - | - | 0.7638 | - | 0.8446 | Exon 11 | Non-Syn. | Arg 46 Lys |
| g.229 T>A | (#) | A | | - | - | - | 16.67 | 3.33 | - | - | - | 33.33 | 6.67 | - | 0.8938 | 0.9234 |  | Non-Syn. | Val 47 Glu |
| g.270 T>G |  | G | | 15.38 | 37.50 | 6.25 | - | 13.33 | 15.38 | 25.00 | 12.50 | - | 13.33 | 0.1402 | 0.1013 | 0.0275 | Intron 11 |  |  |
| g.271 T>A |  | A | | 4.55 | - | 6.25 | - | 3.85 | 9.09 | - | 12.50 | - | 7.69 | 0.0320 | 0.0190 | 0.0015 |  |  |  |
| g.271 T>G |  | G | | 12.50 | 37.50 | - | - | 10.71 | 8.33 | 25.00 | - | - | 7.14 | 0.8745 | 0.8853 | 0.8313 |  |  |  |
| g.274 G>T |  | T | | 3.85 | - | 6.25 | - | 3.33 | 7.69 | - | 12.50 | - | 6.67 | 0.8853 | 0.8938 | 0.8446 |  |  |  |
| g.280 G>A |  | A | | 12.50 | 37.50 | - | - | 10.00 | 8.33 | 25.00 | - | - | 6.67 | 0.0320 | 0.0147 | 0.0012 |  |  |  |
| g.281 G>C | (#) | C | | - | - | 6.25 | - | 3.33 | - | - | 12.50 | - | 6.67 | - | 0.8938 | 0.9204 |  |  |  |
| g.283 delC |  | deletion | | 9.09 | 25.00 | - | - | 6.67 | 0 | 0 | - | - | 0 | 0.0009 | 0.0001 | 0.0000 |  |  |  |
| g.284 T>C | (*) | C | | 4.55 | - | - | - | - | 9.09 | - | - | - | - | 0.8745 | - | 0.9204 |  |  |  |
| g.299 G>A | (*) | A | | 4.55 | - | - | - | - | 9.09 | - | - | - | - | 0.8745 | - | 0.9204 |  |  |  |
| g.311 G>A |  | A | | 45.45 | 37.50 | 43.75 | 33.33 | 46.67 | 72.73 | 75.00 | 87.50 | 66.67 | 80.00 | 0.1217 | 0.0187 | 0.0052 |  |  |  |
| g.327 T>G |  | G | | 40.91 | 50.00 | 31.25 | 50.00 | 40.00 | 81.82 | 50.00 | 62.50 | 100.00 | 66.67 | 0.0217 | 0.1320 | 0.0083 |  |  |  |
| g.335 C>T |  | T | | 13.64 | 12.50 | 25.00 | - | 16.67 | 27.27 | 25.00 | 25.00 | - | 20.00 | 0.6005 | 0.2781 | 0.5623 |  |  |  |
| g.348 G>A |  | A | | 9.09 | 25.00 | - | - | 6.67 | 0 | 0 | - | - | 0 | 0.0009 | 0.0001 | 0.0000 | Intron 12 |  |  |
| g.355 G>A | (# a) | A | | - | - | 18.75 | 33.33 | 16.67 | - | - | 37.50 | 0 | 20.00 | - | 0.2781 | 0.0865 |  |  |  |
| g. 921 G>C | (*) | C | | 3.57 | - | - | - | - | 7.14 | - | - | - | - | 0.8898 | - | 0.9296 |  |  |  |
| g.1025 T>C |  | C | | 10.71 | 37.50 | 12.50 | - | 15.79 | 7.14 | 25.00 | 25.00 |  | 21.05 | 0.0190 | 0.3638 | 0.0404 | Exon 13 | Syn. |  |
| g.1128 T>C |  | C | | 3.57 | - | 9.09 | - | 5.56 | 7.14 | - | 18.18 | - | 11.11 | 0.8898 | 0.8029 | 0.7809 |  | Syn. |  |
| g.1353 T>C |  | C | | 20.83 | 37.50 | 8.33 | - | 15.38 | 25.00 | 25.00 | 16.67 | - | 15.38 | 0.4017 | 0.1402 | 0.1069 | Intron 14 |  |  |
| g.1477 G>A |  | A | | 5.56 | 37.50 | - | - | 13.64 | 11.11 | 25.00 | - | - | 9.09 | 0.8599 | 0.0417 | 0.0469 |  |  |  |
| g.1754 A>C | (#) | C | | - | 12.50 | 25.00 | - | 13.64 | - | 25.00 | 50.00 | - | 27.27 | - | 0.6005 | 0.7245 |  |  |  |
| g.1797 C>T | (*) | T | | 8.33 | - | - | - | - | 16.67 | - | - | - | - | 0.7528 | - | 0.8274 |  |  |  |
| g.1880 C>T | (*) | T | | 8.33 | - | - | - | - | 16.67 | - | - | - | - | 0.7528 | - | 0.8274 |  |  |  |
| g.1914 G>C | (# a g) | C | | - | 37.50 | 25.00 | - | 22.73 | - | 75.00 | 50.00 | - | 45.45 | - | 0.3293 | 0.5476 |  |  |  |
| g.1927 G>C | (#) | C | | - | 12.50 | - | - | 4.55 | - | 25.00 | - | - | 9.09 | - | 0.8745 | 0.9151 |  |  |  |
| g.1994 del A |  | deletion | | 33.33 | 25.00 | 33.33 | 33.33 | 46.15 | 0 | 0 | 0 | 0 | 0 | 0.0005 | 0.0003 | 0.0000 |  |  |  |
| g.2037 G>C | (# a g) | C | | - | 25.00 | 16.67 | 16.67 | 19.23 |  | 50.00 | 33.33 | 33.33 | 38.46 |  | 0.3906 | 0.5875 | Exon 15 | Non-Syn. | Ala205 Pro |
| g.2041 A>C | (# a g) | C | | - | 37.50 | 33.33 | 83.33 | 46.15 | - | 25.00 | 66.67 | 33.33 | 46.15 | - | 0.7968 | 0.0743 |  | Non-Syn. | His 206 Pro |
| g.2065 T>C | (# a g) | C | | - | 25.00 | 25.00 | 33.33 | 26.92 | - | 50.00 | 50.00 | 66.67 | 53.85 | - | 0.1841 | 0.4157 |  | Non-Syn. | Val 214 Ala |
| **All Sequence Variants** | |  | | **9.13** | **18.64** | **11.76** | **11.54** | **13.44** | **12.77** | **20.75** | **20.41** | **12.82** | **16.98** |  |  |  |  |  |  |

**Footnotes:** (*) 6 SVs only in normal samples; (#) 9 SVs only in mammary lesions samples; (a) 5 SVs with significant values of allelic association with the CMLs; (g) SVs with significant values of genotypic association with the CMLs.

**Legend:** A total of 30 SVs were detected with dissimilar observation of 15 SVs in normal and CML samples. The minor allele and the genotype heterozygosis frequencies (%) were calculated individually for each SV. The Hardy-Weinberg equilibrium is indicated by the frequency value (p-value) calculated by the Chi-square test. The SVs that present p < 0.05 are considered to present HWE deviation. The probable amino-acid changes are indicated whit the respective number of the amino-acid in the cat erbB-2_10/15 protein sequence. Two synonymous (Syn.) and five non-Synonymous (Non-Syn.) variations were detected in exonic positions. The zero percentage values were removed for better visualization.

Word (doc.); paper size: 29 x 25 cm.
