# Supplementary material for: ERBB2 in Cat Mammary Neoplasias Disclosed a Positive Correlation between RNA and Protein Low Expression Levels: A Model for erbB-2 Negative Human Breast Cancer
Source: PLoS One. 2013 Dec 26;8(12):e83673. doi: 10.1371/journal.pone.0083673 (PMC3873372; doi:10.1371/journal.pone.0083673)
Supplement: Table S4 — Resume of the AA change effects in cat erbB-2 protein: results of the analysis in Pymol and Polyphen/Polyphen-2 software's. (nc) no changes detected. The categorization results obtained with Polyphen and Polyphen-2 bioinformatics automatic tools are the same. Word (.doc); paper size A4. (DOC) [file pone.0083673.s009.doc]

**Additional Table, Santos et al.; Word (doc.); paper size A4**

**Table S4: Resume of the AA change effects in cat erbB-2 protein: results of the analysis in Pymol and Polyphen/Polyphen-2 software’s.**

|  | **Amino-acid changes** | | | | |
| --- | --- | --- | --- | --- | --- |
| **Results** | **Arg46Lys** | **Val47Glu** | **Ala205Pro** | **His206Pro** | **Val214Ala** |
| AA Side chain polarity (WT>Variant) | nc | Non polar > polar | nc | Polar > non polar | nc |
| AA Side chain charge at pH7 (WT>Variant) | nc | Neutral > negative | nc | nc | nc |
| Residue side chain volume difference (Å^3^) | -5 | -2 | 24 | -41 | -52 |
| Other changes | nc | Hydrophobicity change | nc | nc | Disruption of ligand binding site |
| Score values | 0.002 | 0.994 | < 0.0001 | 0.994 | 0.936 |
| Probably effect in protein | Benign | Probably damage | Benign | Probably damage | Probably damage |

**Legend**: (nc) no changes detected. The categorization of the probably effect in protein are obtained with Polyphen and Polyphen-2 bioinformatics automatic tools.
