# Supplementary material for: ERBB2 in Cat Mammary Neoplasias Disclosed a Positive Correlation between RNA and Protein Low Expression Levels: A Model for erbB-2 Negative Human Breast Cancer
Source: PLoS One. 2013 Dec 26;8(12):e83673. doi: 10.1371/journal.pone.0083673 (PMC3873372; doi:10.1371/journal.pone.0083673)
Supplement: Table S5 — Cat erbB-2 immunohistochemical staining results for each antibody used: CBE356, specific for the external domain and CB11, specific for the internal domain. The strength of immunostaining was estimated as negative (0 and +) or positive (++ and +++). (*) 3 normal mammary samples from 2 hyperplasia cases; (a) Tubulopapillary carcinomas with solid, mucinous or different disease stage; (b) Systemic metastasis; (neo-BL) neoplastic benign lesions; (MaLs) primary malign lesions. Word (.doc); paper size: 25/40 cm. (DOCX) [file pone.0083673.s010.docx]

**Additional data, Santos et al. Word (.doc); paper size: 25/40 cm.**

**Table S5: Cat erbB-2 immunohistochemical staining results for each antibody used: CBE356, specific for the external domain and CB11, specific for the internal domain.**

|  | **Percentage (Ratio observed / n total)** | | | | | | | | | | | | | |
| --- | --- | --- | --- | --- | --- | --- | --- | --- | --- | --- | --- | --- | --- | --- |
|  | **CBE356 / Extracellular IHC scores** | | | | | | | **CB11 / Intracellular IHC scores** | | | | | | |
|  | **0** | **+** | | **++** | | **+++** | | **0** | **+** | | **++** | | **+++** | |
| **Histological Type** | 0 | 0 / + | + | + / ++ | ++ | ++ / +++ | +++ | 0 | 0 / + | + | + / ++ | ++ | ++ / +++ | +++ |
| **Normal Samples:** |  |  |  |  |  |  |  |  |  |  |  |  |  |  |
| Normal mammary samples (n=4*) | 25 |  | 25 |  | 25 |  | 25 |  |  |  |  | 25 | 25 | 50 |
|  | (1/4) |  | (1/4) |  | (1/4) |  | (1/4) |  |  |  |  | (1/4) | (1/4) | (2/4) |
| CMLs adjacent normal tissue (n=8) |  |  | 25 |  | 62.5 |  | 12.5 |  |  | 12.5 |  | 37.5 | 12.5 | 37.5 |
|  |  |  | (2/8) |  | (5/8) |  | (1/8) |  |  | (1/8) |  | (3/8) | (1/8) | (3/8) |
| **Benign Lesions (BLs):** |  |  |  |  |  |  |  |  |  |  |  |  |  |  |
| Tubular Hyperplasia | 100 |  |  |  |  |  |  |  |  |  |  | 100 |  |  |
|  | (1/1) |  |  |  |  |  |  |  |  |  |  | (1/1) |  |  |
| Fibroadenomatous Hyperplasia |  |  |  |  |  |  | 100 |  |  |  |  | 50 |  | 50 |
|  |  |  |  |  |  |  | (2/2) |  |  |  |  | (1/2) |  | (1/2) |
| **Total Hyperplasia (n=3)** | 33.3 |  |  |  |  |  | 66.7 |  |  |  |  | 33.3 |  | 66.7 |
|  | (1/3) |  |  |  |  |  | (2/3) |  |  |  |  | (2/3) |  | (1/3) |
| Fibroadenoma (neo-BL; n=1) |  |  |  |  |  |  | 100 |  |  |  |  |  |  | 100 |
|  |  |  |  |  |  |  | (1/1) |  |  |  |  |  |  | (1/1) |
| **Carcinomas (MaLs):** |  |  |  |  |  |  |  |  |  |  |  |  |  |  |
| Tubular | 100 |  |  |  |  |  |  | 100 |  |  |  |  |  |  |
|  | (1/1) |  |  |  |  |  |  | (1/1) |  |  |  |  |  |  |
| Tubular/solid |  |  | 100 |  |  |  |  |  |  |  |  |  |  | 100 |
|  |  |  | (1/1) |  |  |  |  |  |  |  |  |  |  | (1/1) |
| Papillary | 50 |  |  |  |  |  | 50 |  |  |  |  | 100 |  |  |
|  | (1/2) |  |  |  |  |  | (1/2) |  |  |  |  | (2/2) |  |  |
| Cribiform | 50 |  |  |  | 50 |  |  | 50 |  |  |  |  |  | 50 |
|  | (1/2) |  |  |  | (1/2) |  |  | (1/2) |  |  |  |  |  | (1/2) |
| Tubulopapillary | 66.7 |  | 16.7 |  |  |  | 16.7 | 66.7 |  |  | 16.7 | 16.7 |  |  |
|  | (4/6) |  | (1/6) |  |  |  | (1/6) | (4/6) |  |  | (1/6) | (1/6) |  |  |
| Tubulopapillary [a] |  | 20 |  | 20 | 20 | 20 | 20 | 40 |  | 20 |  |  | 20 | 20 |
|  |  | (1/5) |  | (1/5) | (1/5) | (1/5) | (1/5) | (2/5) |  | (1/5) |  |  | (1/5) | (1/5) |
| **Total carcinomas (n=17)** | 41.2 | 5.9 | 11.8 | 5.9 | 11.8 | 5.9 | 17.7 | 47.0 |  | 5.9 | 5.9 | 17.7 | 5.9 | 17.7 |
|  | (7/17) | (1/17) | (2/17) | (1/17) | (2/17) | (1/17) | (3/17) | (8/17) |  | (1/17) | (1/17) | (3/17) | (1/17) | (3/17) |
| **Metastasis (MeL):** |  |  |  |  |  |  |  |  |  |  |  |  |  |  |
| Lymph node Metastasis | 25 |  | 50 |  |  |  | 25 | 25 |  | 25 |  | 25 |  | 25 |
|  | (1/4) |  | (2/4) |  |  |  | (1/4) | (1/4) |  | (1/4) |  | (1/4) |  | (1/4) |
| Systemic Metastasis [b] | 12.5 |  | 87.5 |  |  |  |  | 37.8 |  | 25.0 |  | 12.5 | 12.5 | 12.5 |
|  | (1/8) |  | (7/8) |  |  |  |  | (3/8) |  | (2/8) |  | (1/8) | (1/8) | (1/8) |
| **Total metastasis (n=12)** | 16.7 |  | 75.0 |  |  |  | 8.33 | 33.3 |  | 25.0 |  | 16.7 | 8.3 | 16.7 |
|  | (2/12) |  | (9/12) |  |  |  | (1/12) | (4/12) |  | (3/12) |  | 2/12 | (1/12) | (2/12) |
|  |  | | |  | | | |  | | |  | | | |
| **RESUME** | **0** | **+** | | **++** | | **+++** | | **0** | **+** | | **++** | | **+++** | |
| Percentage (Ratio observed / n total) | **CBE erbB-2 Negative** | | | **CBE erbB-2 Positive** | | | | **CB11 erbB-2 Negative** | | | **CB11 erbB-2 Positive** | | | |
| **Total normal samples (n=12*)** | 33.3 (4/12) | | | 66.7 (8/12) | | | | 8.3 (1/12) | | | 91.7 (11/12) | | | |
| **Total benign lesions (n=4)** | 25 (1/4) | | | 75 (3/4) | | | | 0 | | | 100 (4/4) | | | |
| Hyperplasias (n=3) | 33.3 (1/3) | | | 66.7 (2/3) | | | | 0 | | | 100 (3/3) | | | |
| Benign neoplasia (n=1) | 0 | | | 100 (1/1) | | | | 0 | | | 100 (1/1) | | | |
| **Total malignant lesions (n=17)** | 58.82 (10/17) | | | 41.17 (7/17) | | | | 52.97 (9/17) | | | 47.06 (8/17) | | | |
| **Total metastatic lesions (n=12)** | 91.67 (11/12) | | | 8.33 (1/12) | | | | 53.33 (7/12) | | | 41.66 (5/12) | | | |
| Lymph node metastasis (n=4) | 75 (3/4) | | | 25 (1/4) | | | | 50 (2/4) | | | 50 (2/4) | | | |
| Systemic metastasis (n=8) | 100 (8/8) | | | 0 | | | | 62.5 (5/8) | | | 37.5 (3/8) | | | |
| **Total lesions samples (n=33)** | 66.67 (22/33) | | | 33.33 (11/33) | | | | 77.27 (16/33) | | | 51.51 (17/33) | | | |

**Legend:** The strength of immunostaining was estimated as negative (0 and +) or positive (++ and +++). (*) 3 normal mammary samples from 2 hyperplasia cases; (a) Tubulopapillary carcinomas with solid, mucinous or different disease stage; (b) Systemic metastasis; (neo-BL) neoplastic benign lesions; (MaLs) primary malign lesions.
