# Supplementary material for: ERBB2 in Cat Mammary Neoplasias Disclosed a Positive Correlation between RNA and Protein Low Expression Levels: A Model for erbB-2 Negative Human Breast Cancer
Source: PLoS One. 2013 Dec 26;8(12):e83673. doi: 10.1371/journal.pone.0083673 (PMC3873372; doi:10.1371/journal.pone.0083673)
Supplement: Table S6 — Correlation measure between ERBB2 RNA and erbB-2 protein expression levels and clinicopathological features of primary mammary lesions. (Red *) Correlation is significant at the 0.05 level. (Red **) Correlation is significant at the 0.01 level. (Corr.) Correlation tests Output. (N) Number of rows in working input data file. (-) Negative relationship between variables. Two-tailed bivariate correlation analysis calculated with indication of two significance levels (p-value not showed). The correlation significant values of the Pearson's analysis were confirmed by the Spearman's test. Word (.doc); paper size A4. (DOC) [file pone.0083673.s011.doc]

**Additional Table, Santos et al.; Word (doc.); paper size A4**

**Table S6: Correlation measure between *ERBB2* RNA and erbB-2 protein expression levels and clinicopathological features of primary mammary lesions.**

| **Correlation testes** | | **Pearson Correlation** | | | | **Spearman's** | | | |
| --- | --- | --- | --- | --- | --- | --- | --- | --- | --- |
| **Expression quantification** | | **Protein** | | **RNA** | | **Protein** | | **RNA** | |
|  | **Correlation Tests Output** | **CB11 internal** | **CBE External** | **Conc_ratio X_100** | **Sample/normal ratio** | **CB11 internal** | **CBE External** | **Conc_ratio X_100** | **Sample/normal ratio** |
| **Clinicopathological features** | |  |  |  |  |  |  |  |  |
| **Tumor Class** | Corr. | -,421 | -,258 | -,411 | -,411 | -,446**^*^** | -,281 | -,299 | -,299 |
|  | N. | 21 | 21 | 18 | 18 | 21 | 21 | 18 | 18 |
| **Age** | Corr. | ,032 | -,104 | -,137 | -,137 | ,129 | -,108 | -,118 | -,118 |
|  | N. | 19 | 19 | 16 | 16 | 19 | 19 | 16 | 16 |
| **lesion size** | Corr. | -,004 | ,000 | -,048 | -,048 | ,014 | -,002 | -,140 | -,140 |
|  | N. | 21 | 21 | 18 | 18 | 21 | 21 | 18 | 18 |
| **mitosis / field** | Corr. | -,387 | -,178 | -,003 | -,003 | -,330 | -,221 | -,030 | -,030 |
|  | N. | 16 | 16 | 14 | 14 | 16 | 16 | 14 | 14 |
| **pleomorphism** | Corr. | -,234 | -,551**^*^** | -,240 | -,240 | -,241 | -,590**^*^** | -,259 | -,259 |
|  | N. | 14 | 14 | 13 | 13 | 14 | 14 | 13 | 13 |
| **Lesions number** | Corr. | ,350 | ,362 | ,300 | ,300 | ,364 | ,361 | ,340 | ,340 |
|  | N. | 20 | 20 | 17 | 17 | 20 | 20 | 17 | 17 |
| **Lynfatic invasion** | Corr. | -,450 | -,269 | -,264 | -,264 | -,422 | -,299 | -,261 | -,261 |
|  | N. | 12 | 12 | 10 | 10 | 12 | 12 | 10 | 10 |
| **Vascular infiltration** | Corr. | -,177 | -,125 | -,050 | -,050 | -,161 | -,065 | -,051 | -,051 |
|  | N. | 13 | 13 | 12 | 12 | 13 | 13 | 12 | 12 |
| **Necrosis** | Corr. | -,313 | -,403 | -,440 | -,440 | -,270 | -,391 | -,278 | -,278 |
|  | N. | 19 | 19 | 16 | 16 | 19 | 19 | 16 | 16 |
| **Clinical Outcome** | Corr. | -,390 | -,247 | -,609**^*^** | -,609**^*^** | -,286 | -,224 | -,563**^*^** | -,563**^*^** |
|  | N. | 17 | 17 | 16 | 16 | 17 | 17 | 16 | 16 |
| **Expression quantification** |  |  |  |  |  |  |  |  |  |
| **IHC CB11 internal** | Corr. | **1** | ,493**^*^** | ,531**^*^** | ,531**^*^** | **1** | ,469**^*^** | ,524**^*^** | ,524**^*^** |
|  | N. | 18 | 21 | 17 | 17 | 18 | 21 | 17 | 17 |
| **IHC CBE External** | Corr. |  | **1** | ,259 | ,259 |  | **1** | ,117 | ,117 |
|  | N. |  | 21 | 17 | 17 |  | 21 | 17 | 17 |
| **RNA Conc_ratio_ X_100** | Corr. |  |  | **1** | 1,0**^**^** |  |  | **1** | 1**,0^**^** |
|  | N. |  |  | 18 | 18 |  |  | 18 | 18 |

**Legend:** (Red *****) Correlation is significant at the 0.05 level. (Red ******) Correlation is significant at the 0.01 level. (Corr.) Correlation tests Output. (N) Number of rows in working input data file. (-) Negative relationship between variables. Two-tailed bivariate correlation analysis calculated with indication of two significance levels (p-value not showed). The correlation significant values of the Pearson's analysis were confirmed by the Spearman’s test.
