# Supplementary material for: ERBB2 in Cat Mammary Neoplasias Disclosed a Positive Correlation between RNA and Protein Low Expression Levels: A Model for erbB-2 Negative Human Breast Cancer
Source: PLoS One. 2013 Dec 26;8(12):e83673. doi: 10.1371/journal.pone.0083673 (PMC3873372; doi:10.1371/journal.pone.0083673)
Supplement: Table S7 — Correlation measure between cat ERBB2 _10–15 Sequence Variants detected and clinicopathological features of cat mammary lesions. Two-tailed bivariate correlation analysis calculated with indication of two significance levels (p-value not showed). The significant values of the Pearson's correlation analysis were confirmed by the Spearman's test. (*) Correlation is significant at the 0.05 level. (**) Correlation is significant at the 0.01 level. (-) negative correlation between the variables. (a) Cannot be computed because at least one of the variables is constant. Word (.doc); paper size 40×20 cm. (DOC) [file pone.0083673.s012.doc]

**Additional Table, Santos *et al*.; Word (doc.); paper size 40x20cm**

**Table S7: Correlation measure between cat *ERBB2*_10-15 Sequence Variants detected and clinicopathological features of cat mammary lesions.**

| **Correlations** | Pearson Correlation | **g.226G>A** | **g.229T>A** | **g.270T>G** | **g.271T>G** | **g.271T>A** | **g.274G>T** | **g.280G>A** | **g.281G>C** | **g.283delC** | **g.284 T>C** | **g.299 A>G** | **g.311 G>A** | **g.327 T>G** | **g.335 C>T** | **g.348 G>A** | **g.355 G>A** | **g. 921 G>C** | **g.1025 T>C** | **g.1128 T>C** | **g.1353 T>C** | **g.1477 G>A** | **g.1754A>C** | **g.1797C>T** | **g.1880C>T** | **g.1914 G>C** | **g.1927 G>C** | **g.1994 de A** | **g.2037G>C** | **g.2041A>C** | **g.2065T>C** |
| --- | --- | --- | --- | --- | --- | --- | --- | --- | --- | --- | --- | --- | --- | --- | --- | --- | --- | --- | --- | --- | --- | --- | --- | --- | --- | --- | --- | --- | --- | --- | --- |
| **tumor Class** | C.C. | .a | 0,296 | -0,280 | -0,311 | 0,000 | 0,035 | -0,312 | 0,035 | -0,226 | .a | .a | -0,020 | 0,072 | -0,146 | -0,226 | 0,291 | .a | -0,241 | 0,040 | -0,362 | -0,427 | -0,249 | .a | .a | -,626* | -0,482 | -0,292 | -0,321 | 0,177 | 0,011 |
|  | N | 15 | 15 | 15 | 14 | 13 | 15 | 15 | 15 | 15 | 15 | 15 | 15 | 15 | 15 | 15 | 15 | 19 | 19 | 19 | 13 | 11 | 11 | 11 | 11 | 11 | 11 | 13 | 13 | 13 | 13 |
| **Age (Years)** | C.C. | .a | .a | 0,114 | -0,127 | 0,298 | 0,314 | -0,164 | 0,314 | .a | .a | .a | ,712* | 0,064 | -0,252 | .a | -0,069 | .a | -0,135 | -0,085 | 0,146 | 0,146 | 0,000 | .a | .a | 0,316 | 0,400 | 0,420 | -0,041 | -0,231 | 0,386 |
|  | N | 8 | 8 | 8 | 7 | 7 | 8 | 8 | 8 | 8 | 8 | 8 | 8 | 8 | 8 | 8 | 8 | 12 | 12 | 12 | 6 | 6 | 6 | 6 | 6 | 6 | 6 | 7 | 7 | 7 | 7 |
| **lesion size** | C.C. | .a | .a | 0,408 | ,801* | -0,258 | -0,267 | ,802* | -0,267 | .a | .a | .a | 0,000 | 0,535 | -0,127 | .a | 0,000 | .a | 0,131 | -0,415 | 0,586 | 0,586 | -0,158 | .a | .a | -0,447 | -0,400 | 0,113 | -0,510 | -0,745 | -0,510 |
|  | N | 8 | 8 | 8 | 7 | 7 | 8 | 8 | 8 | 8 | 8 | 8 | 8 | 8 | 8 | 8 | 8 | 12 | 12 | 12 | 6 | 6 | 6 | 6 | 6 | 6 | 6 | 7 | 7 | 7 | 7 |
| **mitosis / field** | C.C. | .a | .a | .a | .a | .a | .a | .a | .a | .a | .a | .a | .a | .a | .a | .a | .a | .a | -0,447 | -0,316 | .a | .a | .a | .a | .a | .a | .a | .a | .a | .a | .a |
|  | N | 3 | 3 | 3 | 2 | 3 | 3 | 3 | 3 | 3 | 3 | 3 | 3 | 3 | 3 | 3 | 3 | 6 | 6 | 6 | 2 | 2 | 2 | 2 | 2 | 2 | 2 | 3 | 3 | 3 | 3 |
| **Nuclear / cellular pleomorphism** | C.C. | .a | .a | -1,000** | .a | -1,000** | -1,000** | .a | -1,000** | .a | .a | .a | .a | 0,500 | 0,500 | .a | -0,500 | .a | -,913* | -,913* | .a | .a | .a | .a | .a | .a | .a | -1,000** | -0,500 | -0,500 | -0,500 |
|  | N | 3 | 3 | 3 | 2 | 3 | 3 | 3 | 3 | 3 | 3 | 3 | 3 | 3 | 3 | 3 | 3 | 5 | 5 | 5 | 2 | 2 | 2 | 2 | 2 | 2 | 2 | 3 | 3 | 3 | 3 |
| **lesions number** | C.C. | .a | .a | 0,346 | 0,307 | 0,250 | 0,194 | 0,271 | 0,194 | 0,194 | .a | .a | 0,000 | 0,060 | -0,510 | 0,194 | -0,241 | .a | 0,327 | 0,196 | 0,459 | 0,417 | 0,067 | .a | .a | 0,467 | 0,293 | ,655* | 0,089 | 0,034 | 0,218 |
|  | N | 11 | 11 | 11 | 10 | 9 | 11 | 11 | 11 | 11 | 11 | 11 | 11 | 11 | 11 | 11 | 11 | 15 | 15 | 15 | 9 | 8 | 8 | 8 | 8 | 8 | 8 | 10 | 10 | 10 | 10 |
| **Lynfatic invasion** | C.C. | .a | .a | .a | .a | .a | .a | .a | .a | .a | .a | .a | 0,333 | 1,000** | 0,522 | .a | -0,577 | .a | -0,548 | -0,354 | 0,500 | .a | 1,000** | .a | .a | 1,000** | .a | .a | 1,000** | -1,000** | 1,000** |
|  | N | 4 | 4 | 4 | 4 | 4 | 4 | 4 | 4 | 4 | 4 | 4 | 4 | 4 | 4 | 4 | 4 | 7 | 7 | 7 | 3 | 2 | 2 | 2 | 2 | 2 | 2 | 2 | 2 | 2 | 2 |
| **Vascular infiltration** | C.C. | .a | .a | -0,447 | .a | -0,447 | -0,447 | .a | -0,447 | .a | .a | .a | 0,447 | 0,707 | ,894* | .a | -0,707 | .a | -0,258 | -0,577 | -0,577 | -0,577 | 0,577 | .a | .a | 0,000 | -0,577 | -0,667 | -0,167 | -0,612 | -0,167 |
|  | N | 6 | 6 | 6 | 5 | 6 | 6 | 6 | 6 | 6 | 6 | 6 | 6 | 6 | 6 | 6 | 6 | 8 | 8 | 8 | 4 | 4 | 4 | 4 | 4 | 4 | 4 | 5 | 5 | 5 | 5 |
| **Necrosis** | C.C. | .a | .a | -0,447 | .a | -0,447 | -0,447 | .a | -0,447 | .a | .a | .a | 0,447 | 0,333 | 0,447 | .a | 0,000 | .a | -0,316 | -0,598 | -0,612 | -0,612 | -0,167 | .a | .a | -0,667 | -0,612 | -0,707 | -0,707 | -0,577 | -0,707 |
|  | N | 6 | 6 | 6 | 5 | 6 | 6 | 6 | 6 | 6 | 6 | 6 | 6 | 6 | 6 | 6 | 6 | 9 | 9 | 9 | 5 | 5 | 5 | 5 | 5 | 5 | 5 | 6 | 6 | 6 | 6 |
| **Clinical Outcome** | C.C. | .a | .a | -0,772 | -0,772 | .a | .a | -0,772 | .a | .a | .a | .a | 0,343 | -0,275 | 0,642 | .a | 0,086 | .a | -0,361 | .a | -0,772 | -0,772 | 0,140 | .a | .a | 0,140 | .a | -0,772 | 0,140 | -0,046 | 0,140 |
|  | N | 5 | 5 | 5 | 5 | 4 | 5 | 5 | 5 | 5 | 5 | 5 | 5 | 5 | 5 | 5 | 5 | 7 | 7 | 7 | 5 | 5 | 5 | 5 | 5 | 5 | 5 | 5 | 5 | 5 | 5 |

**Legend:** Two-tailed bivariate correlation analysis calculated with indication of two significance levels (p-value not showed). The significant values of the Pearson's correlation analysis were confirmed by the Spearman’s test. (*) Correlation is significant at the 0.05 level. (**) Correlation is significant at the 0.01 level. (-) negative correlation between the variables. (a) Cannot be computed because at least one of the variables is constant.
